# Programmable Microcarriers for Stem Cell Therapy: Advanced Fabrication Strategies, Stem Cell Fate Regulatory Function and Biomedical Applications

**DOI:** 10.3390/ijms27135784

**Published:** 2026-06-26

**Authors:** Yuqi Wang, Changmin Hu

**Affiliations:** 1 College of Life Science and Technology, Huazhong Agricultural University, No.1 Shizishan Street, Hongshan District, Wuhan 430070, China; wangyuqi@webmail.hzau.edu.cn; 2 College of Veterinary Medicine, Huazhong Agricultural University, No.1 Shizishan Street, Hongshan District, Wuhan 430070, China

**Keywords:** programmable microcarrier, stem cell, microfluidics, cell fate regulation, stemness maintenance, stem cell therapeutic applications

## Abstract

Stem cells, with their self-renewal and multi-lineage differentiation potential, hold promise for tissue repair and intractable diseases treatment. Yet clinical translation of stem cell therapies has long been hindered by insufficient scalable stem cell manufacturing, stemness loss and functional decline in 2D expansion, and poor post-transplantation cell retention, unregulated fate control. Programmable microcarriers (MCs) paired with 3D dynamic culture offer an emerging strategy to address these bottlenecks and enable stem cell fate regulation. In this review, we systematically review advanced MC fabrication strategies for stem cell fate regulation, comparing features of emerging technologies (microfluidics, electrospraying, in-air microfluidics, integrated in situ functionalization) and their implications for programmable MC control and scalable manufacturing. We analyze how MCs modulate stem cell behaviors (adhesion, proliferation, stemness maintenance, differentiation) via synergistic static physicochemical cues and dynamic stimuli-responsive properties. We map the latest advances in functionalized MC-mediated stem cell therapy across osteochondral defects, autoimmune, skin, ophthalmic and neurodegenerative diseases. Finally, we pinpoint unresolved challenges for clinical translation of MC–stem cell system and outline key future research directions. This review offers a systematic roadmap for advancing programmable MC fabrication, clinical-grade stem cell biomanufacturing, and precise cell therapy development.

## 1. Introduction

Cell-based therapy has demonstrated enormous potential for neotissue regeneration and treating various diseases, which aims at restoring injured tissue or organ to their original condition by transplanting therapeutic cells to the affected site and replacing the damaged ones [1]. Stem cells (SCs) are primitive cells characterized by self-renewal capacity and multi-lineage differentiation potential. Under specific conditions, they can undergo self-renewal to generate identical daughter SCs, or differentiate into functionally specialized mature somatic cells through multi-directional differentiation. SCs exert therapeutic effects either by directly differentiating into functional cells to replace damaged tissues, or through paracrine mechanisms involving the secretion of growth factors and cytokines to modulate the microenvironment, promote neovascularization, and suppress inflammatory responses [2]. They have demonstrated remarkable clinical efficacy in a broad spectrum of refractory diseases, including hematological disorders, cardiovascular diseases, neurodegenerative diseases, autoimmune diseases, osteoarticular injuries, and organ failure. SCs not only extend beyond providing novel therapeutic strategies for diseases that are recalcitrant to conventional medical interventions, but also offer breakthrough prospects for addressing healthcare challenges such as organ donor shortage and the increasing incidence of degenerative diseases.

**Figure 1:**
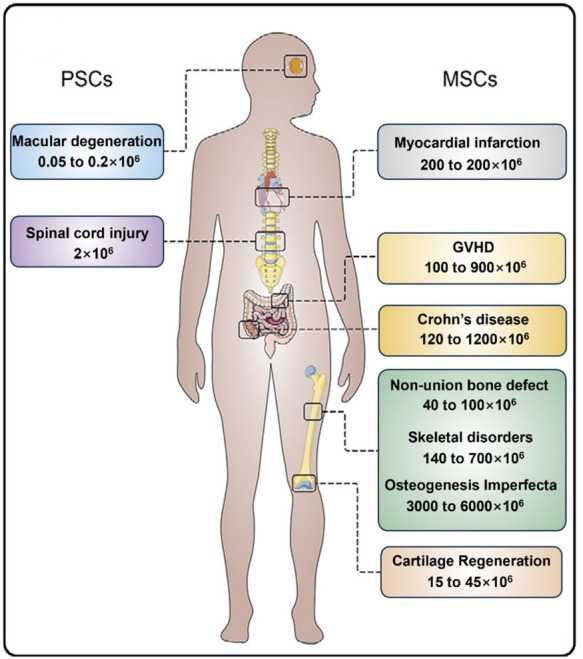
Schematic representation of required number of cells for some clinical therapies via stem cells. Reproduced with permission [4,5]. 2013, Elsevier.

In fact, the estimated stem cell dose required per patient varies across diseases, with tens of millions to billions of MSCs or PSCs typically needed per kilogram of body weight, as shown in Figure 1 [3,4,5,6]. However, SCs derived from the muscle, and dental pulp are limited by low cell abundance and high difficulty in isolation and purification. In addition, insufficient efficiency of in vitro large-scale expansion remains a common challenge for the clinical translation of all types of SCs. Unlike chemical drugs or biological products, SCs are “living products,” and their manufacturing process necessitates the maintenance of cell viability, phenotypic stability, and functional properties. Furthermore, long-term two-dimensional (2D) culture of SCs leads to the alteration of cell extracellular matrix (ECM) secretion, loss of specific morphology, and compromised differentiation capacity, homing ability, immunomodulatory function and paracrine function [7]. These challenges contribute to persistently high production costs for SC therapy products and make quality consistency difficult to ensure, severely constraining their commercialization process and patient accessibility. More importantly, the growth and differentiation of SCs require a stem cell niche analogous to native tissues, which constitutes a specialized microenvironment formed by the coordinated interplay of growth factors, cytokines, surface-bound signaling molecules, cell–cell contacts, extracellular matrix, and local biomechanical cues [8].

Microcarriers (MCs) are spherical particles typically 100–300 μm in diameter that provide a scaffold for cell growth by mimicking the three-dimensional (3D) microenvironment in vivo, representing a pivotal technology for 3D cell culture [9]. By suspending microcarriers in culture medium, adherent-dependent SCs can attach, spread, and expand efficiently on their surfaces, transforming the culture system from 2D planar configurations to 3D spatial architectures and substantially enhancing SC yield per unit volume and overall culture efficiency [10]. Furthermore, the selection of microcarrier materials (such as dextran, cellulose, gelatin, etc.) and their surface functionalization modifications (functional groups, pore structures, topography, roughness, etc.) can precisely emulate the biochemical properties of microcarriers, providing appropriate biomechanical signals and adhesion sites for SCs, thereby better maintaining their undifferentiated state, self-renewal capacity, and multi-lineage differentiation potential. Evidently, microcarrier-based 3D culture technology serves as a critical breakthrough for SCs in vitro expansion and holds significant importance for achieving scalable manufacturing of SC therapeutic products.

**Figure 2:**
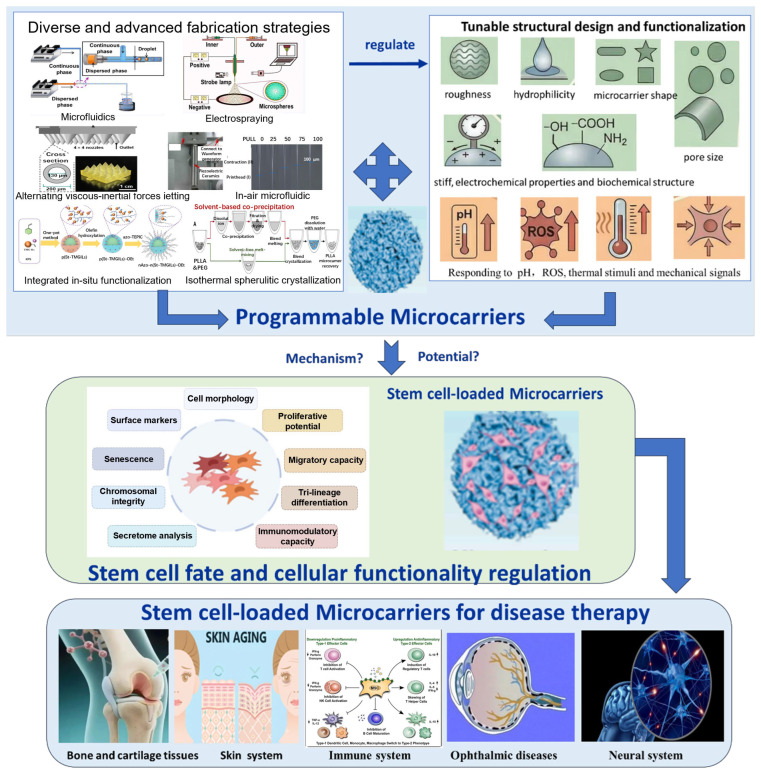
Programmable microcarriers regulate stem cell fate for disease therapy. Diverse and advanced fabrication strategies. Reproduced with permission [11] 2020, Wiley [12]; 2018, Elsevier [13]; 2025, Elsevier [14]; 2021, MyJoVE Corporation [15]; 2018, AAAS [16]; 2023, Elsevier; Tunable structural design and functionalization. Created by Microsoft PowerPoint. Stem cell fate and cellular functionality regulation. Created by Microsoft PowerPoint. Stem cell-loaded microcarriers for disease therapy. Reproduced with permission [17]. 2022, Springer LTD, [18] 2025, Elsevier, [19] 2023, Elsevier, [20] 2025, AAAS, [21] 2015, Elsevier.

In this review, based on recent advances in stem cell-based disease therapy, we systematically elaborate on the advanced fabrication methods of microcarriers (MCs), as well as the synergistic effects of the 3D microenvironment—constructed by static structural parameters (e.g., pore size, curvature, surface roughness, microcarrier shape, stiff, hydrophilicity, electrochemical properties and biochemical structure)—and the dynamic sensing and closed-loop response capabilities of MCs (e.g., sensing and responding to pH fluctuations, changes in reactive oxygen species levels, thermal stimuli, and mechanical signals in the microenvironment) on large-scale stem cell manufacturing, stem cell fate regulation, and cellular functionality. Finally, we present the therapeutic applications of SCs expanded via MC-based 3D culture systems. This review summarizes the latest scientific progress on MCs and their applications in the biomedical field (Figure 2).

## 2. Stem Cells Therapy Potential and the Role of Microcarriers

### 2.1. Stem Cell Therapy Potential

Stem cell technology has opened a new path for the clinical treatment of intractable diseases and organ transplantation, offering renewed hope for many diseases with poor efficacy of traditional treatments [22,23]. Over the past decade, numerous preclinical investigations and early-phase clinical trials worldwide have continuously advanced, supporting the safety and clinical feasibility of various stem cell types, including iPSCs, ESCs, and MSCs [24,25].

To date, multiple allogeneic stem cell therapies have reached commercialization, including Prochymal (Osiris Therapeutics, Columbia, MD, USA), approved in Canada and New Zealand for graft-versus-host disease, and Cartistem (Medipost Co., Ltd., Seoul, Republic of Korea), approved for osteoarthritis treatment. The therapeutic effects of stem cells (SCs) are primarily mediated by three core mechanisms: immunomodulation, paracrine effects, and differentiation into functional cells. SCs exert therapeutic effects in multiple indications including hematopoietic stem cell transplantation for hematological diseases, osteoarthritis, graft-versus-host disease, myocardial infarction, and neurodegenerative diseases, bringing new hope to numerous patients with unmet medical needs [26,27,28,29]. In summary, SCs, with their unique self-renewal ability and multi-directional differentiation potential, play an irreplaceable role in the treatment of intractable diseases, tissue and organ repair, and the field of regenerative medicine.

### 2.2. Advances in the Regulation of Stem Cells by Microcarriers

The earliest application of microcarriers for SC culture can be traced back to the late 1960s, when Van Wezel first developed DEAE-dextran-based MCs for of anchorage-dependent cells [30]. In the 1980s, researchers began exploring microcarrier-based culture technologies to address the challenges of SC in vitro expansion [31]. Early MCs were predominantly dextran-based, such as the Cytodex series, whose favorable biocompatibility and moderate surface charge properties were suitable for SC adhesion and growth. However, these initial MCs did not adequately account for the specialized microenvironmental requirements of SCs, including extracellular matrix mimicking, biomechanical signal transduction, and directed differentiation induction [4].

The escalating demand for 3D cell culture has catalyzed the rapid development and commercialization of MCs. Recently, MC materials have gradually diversified from dextran to various biomaterials including cellulose, GelMA, chitosan, and poly (lactic-co-glycolic acid) (PLGA) [32]. Due to their biocompatibility, biodegradability and non-toxicity, several silk fibroin spherical MCs in combination with alginate, gelatin and calcium phosphates have been reported with very interesting outcomes. In addition, other silk-based three-dimensional structures such as microparticles with chitosan and collagen, as well as organoids, have been increasingly studied [33]. In stirred flasks, Cytodex 1 microcarriers can achieve cGMP-grade expansion of umbilical cord mesenchymal stem cells (UCMSCs), yielding 84 × 10^6^ cells within 6 days with an expansion fold of up to 37 and a harvest efficiency of approximately 95%, while maintaining their phenotype and differentiation potential [34]. The developed serum-free microcarrier-based culture system enables scalable expansion of bone marrow mesenchymal stem cells (BMSCs) and adipose mesenchymal stem cells (ADSCs), with maximum cell densities reaching 3.6 × 10^5^ cells/mL and 1.9 × 10^5^ cells/mL, respectively [35]. Corning^®^ dissolvable microcarriers, combined with serum-free medium, achieved a 16.5-fold proliferation of umbilical cord-derived mesenchymal stem cells (UC-MSCs) within 5 days, with the phenotype of expanded cells fully complying with the criteria established by ISCT [36]. The 3D microcarrier technology realized a more than 200-fold expansion efficiency of amniotic epithelial stem cells (AESCs), far exceeding the 10-fold expansion level of 2D planar culture. Thermoresponsive brush gel MCs enabled a 5.3-fold proliferation of hBM-MSCs in 5 days, with an enzyme-free harvest efficiency of 69% at low temperature and a cell viability over 95%, which effectively avoided cell damage caused by enzymatic digestion [37]. Alginate-gelatin composite biodegradable MCs achieved a 5-fold increase in stem cell proliferation efficiency compared with 2D culture in a 100 L bioreactor system, with a single-batch yield exceeding 100 billion cells and a 37% enhancement in differentiation potential versus conventional methods [38]. Therefore, MCs markedly facilitate the scalable expansion of SCs, exert critical roles in maintaining SC stemness and enhancing the quality of cell products, and hold vital significance for the industrial translation of SC-based therapeutic products.

## 3. Functional Microcarriers for Stem Cell Culture

### 3.1. Design Principles and Classification of Microcarriers

**Figure 3:**
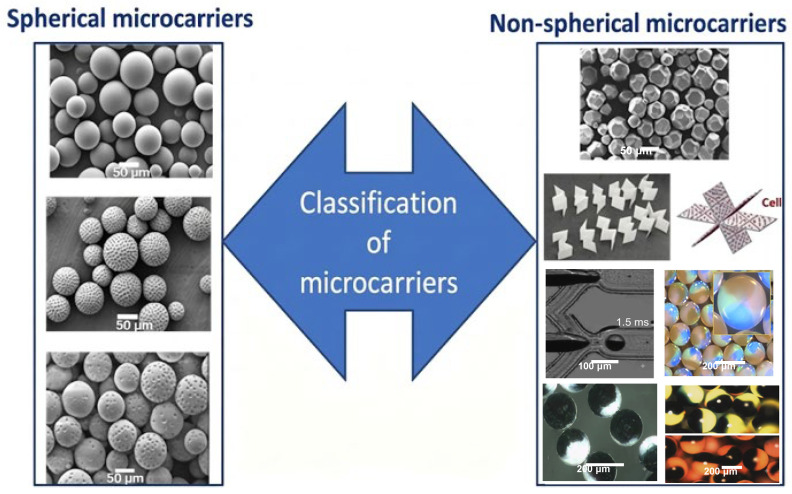
Classification of microcarriers based on their geometric characteristics. Reproduced with permission [39] 2025, Wiley.

Based on their geometric characteristics, the microcarriers applied in the stem cell field can be divided into two major categories: spherical microcarriers and non-spherical microcarriers (Figure 3). Spherical microcarriers include: (1) Solid smooth spherical microcarriers. They are the most widely used commercial type, featuring uniform surface curvature, symmetric hydrodynamic properties, and excellent suspension stability. The representative product is the Cytodex^®^ series. (2) Porous spherical microcarriers. With an interconnected pore structure, they combine the hydrodynamic advantages of spherical geometry with three-dimensional growth space, which facilitates cell migration into the carrier interior and aggregate formation. Representative products include Cytopore^®^ and CultiSpher^®^. (3) Spherical microcarriers with surface micro-topography: Micro/nano structures such as wrinkles, pits, and ridge-like patterns are constructed on the spherical surface to regulate the local surface topology and the distribution of adhesion sites, thereby enabling precise control over cell spreading, cytoskeletal reorganization, and differentiation fate.

Non-spherical microcarriers include: (1) Foldable plate-shaped microcarriers; they have a higher specific surface area than spherical microcarriers of the same volume. However, such structures face challenges including orientation control, shear-induced damage at the edges, and stacking/aggregation during stirred culture. (2) Irregular-shaped microcarriers: These refer to non-spherical geometries including ellipsoidal, polyhedral and other shapes, characterized by heterogeneous curvature distribution and anisotropic structure. (3) Multi-compartment microcarriers; they have complex geometries with core–shell structures, multi-cavity compartments, or coaxial channels, enabling spatially compartmentalized co-culture of multiple cell types or gradient release functions. Representative available commercial MCs are presented in Table 1.

### 3.2. Microcarriers Characteristic for 3D Stem Cell Culture

Microcarriers hold unique advantages for the scalable expansion of SCs. Beyond the inherent high surface-area-to-volume ratio, microcarriers for 3D SC culture should possess the following characteristics:

(1) Tunable particle size distributions, which shortens the diffusion pathways of nutrients and metabolites, thus optimizing mass transfer efficiency;
(2) Capability for continuous passaging via bead-to-bead transfer, providing dynamic growth space for sustained cell proliferation while avoiding cellular damage caused by conventional enzymatic dissociation;
(3) Excellent ability to construct microniches, which recapitulates the compositions and topographical architectures of ECM to preserve stemness phenotypes and promote functional expression;
(4) Highly tunable physicochemical properties and surface functionalization cues, enabling precise customization for cell type-specific requirements;
(5) Compatibility with cell-laden injection, offering direct cell delivery for in situ tissue repair and cell therapy applications;
(6) Robust adaptability to bioreactor systems, facilitating real-time process monitoring and automated culture control.

The above features enable MCs to establish an in vivo-like growth microenvironment for SCs, supporting their efficient adhesion, proliferation and directed differentiation. The physicochemical properties (such as material composition, surface charge, and particle size) and functional modifications (such as surface coating and bioactive factor immobilization) of MCs directly determine the adhesion efficiency, proliferation activity and differentiation potential of SCs. The precise regulation of key biological characteristics of SCs is highly dependent on the advanced MC fabrication strategies of MCs. Innovation in MC preparation processes and surface modification is expected to optimize the structure and performance of MCs, thereby further improving the efficiency of SC expansion and the stability of long-term stemness maintenance.

Collectively, microcarrier-based 3D culture systems integrate the advantages of high-efficiency expansion, quality controllability, and clinical translatability, which is suitable for industrialized stem cell manufacturing.

## 4. Advanced Fabrication Strategies of Programmable Microcarriers for Orchestrated SCs Fate Regulation

Despite the remarkable therapeutic efficacy of SC therapy, its widespread clinical translation is severely limited by the difficulty of large-scale, standardized stem cell manufacturing. This bottleneck is fundamentally rooted in MCs: as core materials supporting SC adhesion, proliferation and stemness maintenance during expansion, their performance directly determines the efficiency, quality and batch-to-batch consistency of SC products, while innovation in their fabrication technology dictates the products’ biosafety and clinical therapeutic efficacy [40]. Conventional MC fabrication methods (mechanical stirring emulsification, suspension polymerization, phase separation, etc.), despite suitability for large-scale production, have inherent flaws including broad size distribution, poor monodispersity, limited microstructure control, toxic additive residues, and uneven surface functionalization, which cannot meet the stringent material requirements for cGMP-compliant clinical-grade cell manufacturing.

**Figure 4:**
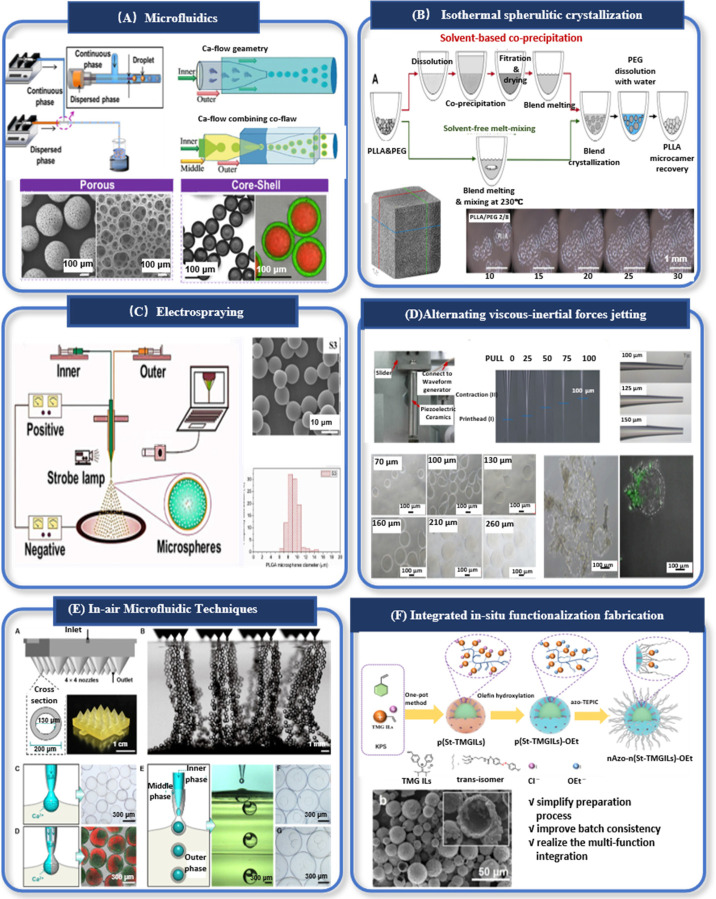
Schematic illustration of typical microcarrier preparation method and their corresponding morphologies. (**A**) Microfluidics. Reproduced with permission. [11] 2020, Wiley. (**B**) Isothermal spherulitic crystallization. Reproduced with permission. [12] 2018, Elsevier. (**C**) Electrospraying, Reproduced with permission. [13] 2025, Elsevier. (**D**) Alternating viscous-inertial forces jetting. Reproduced with permission. [14] 2021, MyJoVE Corporation. (**E**) In-air microfluidic techniques. Reproduced with permission. [15] 2018, AAAS. (**F**) Integrated in situ functionalization fabrication. Reproduced with permission. [16] 2023, Elsevier.

In recent years, multidimensional breakthroughs have been achieved in MC preparation technology, including microfluidics, electrospraying, isothermal spherulitic crystallization, alternating viscous-inertial forces jetting, in-air microfluidic techniques and integrated in situ functionalization fabrication technology (Figure 4). These advances not only overcome the inherent drawbacks of traditional processes from a material science perspective, but also drive the functional transformation of microcarriers from cell expansion to an integrated platform for SC fate regulation and therapy [41].

### 4.1. Microfluidics

Microfluidic technology enables the fabrication of microcarriers with uniform size and controllable morphology through regulation of droplet generation and solidification processes. By essence, microfluidic preparation of MCs is to utilize the laminar flow characteristics and shear effect of multiphase fluids to generate uniform microdroplet templates, which are then solidified in situ through cross-linking [42]. Usually, the disperse phase is a precursor solution containing biocompatible polymers, active factors, or cells, and the continuous phase is an oil phase containing stabilizers. By regulating parameters such as the flow rate ratio of the two phases, fluid viscosity, interfacial tension, and channel structure, MCs can be prepared within the particle size range of 60–300 μm, with a coefficient of variation (CV) of particle size less than 3%, which is far superior to the CV value of more than 20% of the traditional stirring emulsification method. This fundamentally eliminates problems such as differences in cell seeding density, poor growth synchronization, and fluctuations in stemness maintenance caused by uneven MC size [43]. The precursor solution used in this method not only needs to have low viscosity to ensure stable pumping in the microchannels, but also the droplets need to be rapidly cross-linked and solidified to avoid agglomeration and deformation during collection. At present, various solidification methods, such as photo-cross-linking, ionic cross-linking, and phase separation, have been developed, which are suitable for systems such as polyethylene glycol (PEG), gelatin methacryloyl (GelMA), sodium alginate, and chitosan [44].

The main channel configurations of microfluidics include T-type, flow-focusing type, and co-flow type [11,45], which are respectively suitable for the preparation of conventional, high-precision, and core–shell structure MCs. By increasing the number of channel stages, combining the emulsion templating method and droplet inner-phase separation method, MCs with various structures from solid, porous, hollow, core–shell, Janus to complex multi-compartment can be constructed in one step. Among them, core–shell structure MCs can achieve long-term sustained release by loading growth factors in the core, and provide adhesion sites by grafting RGD cell adhesion peptides on the shell, synchronously realizing high-density expansion and directed differentiation regulation of SCs. Relevant studies have confirmed that this type of MC can increase the expansion multiple of human MSCs by more than 40% compared with commercial microcarriers, and can better maintain the multi-directional differentiation potential and paracrine function of cells [46,47]. In addition, the emulsion templating method includes single emulsification and multiple emulsifications [48]. The single emulsification system is simple to operate and has high flux, which can be used to prepare Janus-type or stimulus-responsive MCs. The fluorescent sodium alginate MCs prepared by Chen et al. [49] can realize the tracking of drug delivery. Liu et al. [50] prepared near-infrared-responsive Cur-Fe_3_O_4_@HMP microcarriers synergistically for treating endometriosis. The multiple emulsification templating method uses laminar flow to achieve partitioned solidification of components in droplets, which can prepare core–shell or complex multi-compartment MCs and realize independent encapsulation of multiple components. Zhang et al. [51] prepared double-compartment, triple-compartment, and quadruple-compartment calcium alginate microcarriers, successfully encapsulating MSCs and HUVECs in adjacent compartments, realizing partitioned co-culture of different types of cells. At present, stable preparation of up to five-fold emulsions can be achieved. The droplet inner-phase separation method can form partitioned structures in one step through solvent evaporation or temperature-induced phase separation, which is fixed by photopolymerization, realizing low-cost and site-specific encapsulation of multiple components [29,52].

Although microfluidic technology has significant advantages in precision, it faces two major bottlenecks in large-scale application. First, low production efficiency and high production cost. In the future, it is necessary to develop equipment such as multi-channel parallel microfluidic systems and high-speed surface exposure photocuring 3D printing technology; or improve droplet generation efficiency through special vibration methods, regulate droplet size by combining vibration parameters, and greatly reduce production costs at the same time [40]. Second, there are potential biotoxicity risks of oil phase, cross-linking agents, etc., in the traditional system. In the future, air microfluidic systems can be considered for microcarriers, using gas shear instead of oil phase systems; or through mild visible light cross-linking and oil-free system design, an all-aqueous microfluidic system can be established to completely avoid organic solvent residues, to meet the needs of clinical-grade SC culture systems.

### 4.2. Electrospraying

Electrospray (ES) technology has emerged as a highly promising approach for the laboratory-scale fabrication of microcarriers, distinguished by high monodispersity and mild processing conditions. Mechanistically, the ES process is driven by electrostatic field. Specifically, a high-voltage potential difference is established between the needle and the receiving substrate, which charges the biopolymer solution pumped through the spinneret and induces its deformation into a stable conical geometry known as the Taylor cone. A charged liquid jet is then ejected from the apex of the Taylor cone, which undergoes sequential Rayleigh fission and Coulomb breakup to generate monodisperse micron-sized droplets. These droplets are subsequently solidified in situ to form the final MCs [13].

Notably, the mild processing conditions and precise droplet controllability inherent to ES render it ideally suitable for the encapsulation of living cells, as well as for the processing of electric field-polarizable polymer solutions or suspensions with matched conductivity and surface tension. Furthermore, the molding quality, particle size distribution, and micromorphology of ES-fabricated MCs are predominantly governed by a set of core factors, including the applied voltage, nozzle specifications, auxiliary gas pressure, and physicochemical properties of the polymer feed solution [53,54,55].

Most of the reports revealed that bioelectrospraying is safe and does not implicate any adverse effects in terms of cell viability, morphology, and proliferation that greatly improves the safety and efficacy of the process. Polymers like CS, alginate, polyethylene glycol are more suitable for the bioelectrospraying technique [56].

ES technology was utilized to fabricate genipin cross-linked alginate–chitosan hydrogel microcarriers. Monodisperse alginate beads with uniform microstructure were generated via ES, followed by chitosan coating and genipin cross-linking. MSCs cultured on these microcarriers exhibited 26% higher cell attachment and doubled proliferation rate compared to Cytodex I. Meanwhile, they supported facile cell detachment without prolonged incubation or intense agitation during the cell harvest [57]. In another report, glycosaminoglycan (GAG)–chitosan microcarriers for MSC encapsulation were fabricated by ES. ES yielded highly uniform microcapsules outperforming traditional air atomization. Hyaluronic acid-based capsules showed better cell adhesion than carboxymethyl cellulose counterparts. The results indicated that the electrospraying is a highly efficient, and scalable approach for MSC encapsulation [58,59]. Other study confirmed that highly monodisperse MCs fabricated by ES show a significant negative correlation between particle size and applied voltage, and a positive correlation with nozzle inner diameter [60].

Electrospraying holds significant application potential in large-scale SC expansion, stemness maintenance, and cell therapy, with advantages including precise MC size and morphology control, mild cell-compatible processing, easy scale-up, and an oil-free, demulsification-free workflow that inherently reduces organic solvent residue. However, two key limitations remain: first, electric field interference between multi-needle arrays during scale-up causes inter-batch MC size inconsistency; second, poor processability of high-viscosity, high-cell-density bioinks, with frequent needle clogging and elevated cell damage, narrows its applicable material scope.

Two differentiated technical approaches grounded in the design flexibility of microcarrier systems are expected to address the bottlenecks of needle clogging and compromised cell viability associated with high-cell-density bioinks in electrospraying.

The first approach follows a cell-free fabrication and post-seeding expansion paradigm. Cell-free polymeric microcarriers are first fabricated by electrospraying, after which stem cells are seeded onto the carrier surfaces for adherent culture and expansion in vitro. Crucially, this strategy entirely circumvents process-related cell injury, since stem cells are not exposed to the deleterious conditions of electrospraying—high-voltage electric fields, elevated wall shear stress, and tensile forces arising from jet breakup and droplet formation—at any stage of fabrication. By engineering microcarrier properties across multiple length scales, from bulk material composition and surface chemistry to internal porous microstructure and topographic features, a biomimetic niche is provided that supports robust stem cell adhesion, maintains stemness, and sustains long-term proliferation. High cell densities matching clinical therapeutic requirements can therefore be achieved purely through post-fabrication expansion, with no need for high-density cell loading during the spraying process itself.

The second approach relies on one-step electrospraying of low-to-moderate density cell suspensions, paired with post-fabrication in situ expansion. Here, stem cells are mixed homogenously with the biomaterial matrix at cell densities that fall well within the stable operating window of conventional electrospraying, enabling one-step generation of cell-laden microcarriers. Limiting the initial cell loading in this way avoids the sharp viscosity increase and spontaneous cell aggregation that plague high-density bioink formulations, substantially reducing clogging risk at the nozzle. Rheological properties can be further optimized by adjusting solid content, solid particle morphology, and processing parameters such as applied voltage and feed rate, providing an additional layer of control over both clogging propensity and shear- or electric field-mediated cell damage. Over the culture period, cells both encapsulated within the microcarrier matrix and adherent to its surface proliferate in situ, yielding orders-of-magnitude increases in cell number and ultimately reaching the high doses required for clinical translation.

The two approaches are complementary and suited to distinct translational contexts. The cell-free route is preferable for large-batch production and industrial scale-up, particularly when working with shear-sensitive primary stem cell types. The cell-laden approach, by contrast, retains the simplicity of one-step electrospray encapsulation and offers more homogeneous cell distribution, making it better suited to point-of-care therapeutic settings.

### 4.3. Isothermal Spherulitic Crystallization

Most conventional fabrication methods for porous polymeric microcarriers suffer from reliance on toxic organic solvents, complex equipment with time-consuming drying steps and costly waste management, which severely limit their large-scale manufacturing.

A fully organic solvent-free method was developed to fabricate porous PLLA microcarriers via spherulitic crystallization of PLLA from PLLA/PEG blends. This method enables independent modulation of microcarrier size and porosity via an eco-friendly, biocompatible and easily scalable production, with a higher crystallization temperature leading to a larger size, and a higher PLLA content in the precursor blend resulting in a lower microcarrier porosity. Moreover, they support the long-term proliferation and osteogenic differentiation of human adipose stromal/stem cells (hASC). The microcarriers prepared by this method range from 100 to 230 μm with tunable porosity, but are limited to crystallizable polymers. For future advancement of this technology, the combination of PEG of higher molar mass and extrusion blending process is expected to achieve scale-up production of porous polymers microcarriers [12].

### 4.4. Alternating Viscous-Inertial Forces Jetting

Current methods for preparing microcarriers of microfluidic chips and electrospray highly rely on custom nozzles or fixed configuration flow channels. When adapting to the preparation requirements of various bioinks, biomaterials, and microcarriers with different particle sizes, these methods face critical limitations including high processing difficulty, inconvenient device replacement, poor process regulation, and high fabrication cost, which severely restrict their large-scale biomedical application. Alternating viscous-inertial forces jetting (AVIFJ), a modified 3D jetting printing technology was developed to fabricate hydrogel microcarriers. This technique enables controllable droplet formation by the downward driving force and static pressure generated via vertical vibration to overcome the surface tension at the nozzle tip. Unlike conventional methods requiring harsh mechanical or thermal processing, AVIFJ operates via high-frequency small-amplitude rapid displacements, which negligibly impairs bioink physicochemical properties and bioactivity, making it suitable for various biomaterials. Alginate-collagen microcarriers with tunable diameters of 100–300 μm were successfully fabricated by AVIFJ. The resulting microcarriers provide a favorable growth microenvironment and exhibit excellent cell adhesion performance [14].

For future optimization, satellite droplet removal and external driving force application can further improve the fabrication stability of microcarriers. While external driving forces are expected to expand the type and concentration range of printable inks, the limited driving force of the current printing technology remains a key challenge, especially for processing high-viscosity inks, which will be the core focus of our subsequent technological improvement.

### 4.5. In-Air Microfluidic Techniques

In-air microfluidic techniques have emerged as an alternative to enclosed microfluidic channels, achieving droplet generation via colliding micrometer-scale liquid jets in open air [15]. This method uses a vibrating piezoelectric element to induce the Rayleigh–Plateau instability of a liquid jet from a nozzle, forming droplets that collide with another liquid jet in air to complete encapsulation. An analogous strategy uses a vibrating needle to periodically inject the dispersed phase across a gas–liquid interface, producing emulsions via interfacial shearing. Although these off-chip approaches largely overcome the limitations of enclosed microfluidic channels, they still require complex device fabrication (e.g., integrating piezoelectric element) and precise operational control (e.g., a pulse generator), which greatly hinder their industrial scale-up [61].

**Figure 5:**
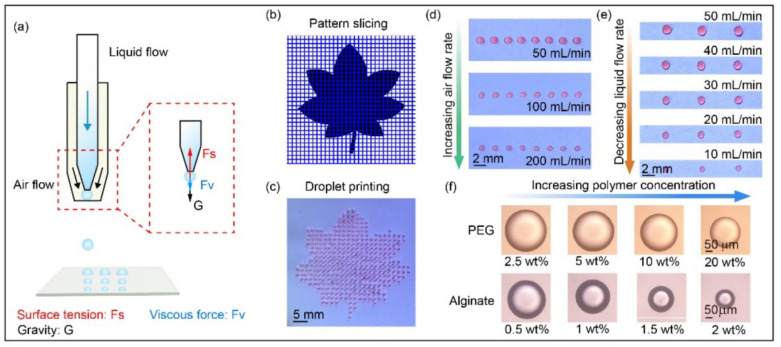
Droplet patterns designed by air-focused microfluidic 3D droplet printing (AFMDP). (**a**) Schematic diagram of AFMDP, in which surface tension held the droplet to the nozzle while gravity and viscous drag pulled the droplet off the nozzle. (**b**) Pattern slicing and (**c**) droplet printing by AFMDP with a modified Gcode. Designed pictures were sliced into droplet patterns were sliced using Ultimaker Cura (version 5.2.1), and each mesh represents one droplet site. Optical images of droplets prepared using (**d**) different air flow rates, (**e**) different liquid flow rates, and (**f**) different polymer concentrations. Reproduced with permission. [62] 2024, Elsevier.

An air-focused microfluidic 3D droplet printing (AFMDP) system, integrating microfluidics and 3D printing, was developed for the facile preparation of droplets with tunable size and controlled position (Figure 5). By using air as the continuous phase, AFMDP enables the fabrication of monodisperse particles via droplet templating, while eliminating the post-preparation oil phase removal step required in conventional emulsion-based systems. For example, droplets of alginate hydrogel were fabricated by cross-linking hydrogel particles in a Ca^2+^-containing collection bath. This was because divalent Ca^2+^ cations could serve as crosslinkers, and each Ca^2+^ cation could simultaneously bind to two carboxylic groups from two different alginate molecules. Similar to droplets, hydrogel particles prepared by AFMDP had a narrow size distribution of 196 ± 5 μm. In addition, the diameter of hydrogel particles could be tuned by varying the inner nozzle diameter, air flow rate, and alginate concentration [62].

In-air microfluidic technology has revolutionized microcarrier manufacturing throughput (a 1000-fold increase) and greatly improved biocompatibility with an oil-free, emulsifier-free workflow, by shifting microfluidic processing from inside a closed chip to open air. The 2024 commercial launch of DMC dissolvable microcarriers marks this technology’s transition from lab research to industrial application, providing an efficient, mild, and scalable manufacturing platform for cell therapy, vaccine manufacturing, and tissue engineering. Its key advantages include the ability to fabricate microcarriers with precise sizes, complex structures, and excellent cytocompatibility. It also simplifies downstream cell harvesting processes, and significantly improves cell recovery rates.

### 4.6. Integrated In Situ Functionalization Fabrication

Conventional MCs are generally subjected to functional modification via secondary grafting, physical adsorption and other post-fabrication processes. This post-modification strategy restricts functional molecules to be modified mostly on the microcarrier surface with uncontrollable distribution uniformity, and suffers from inherent drawbacks including low grafting efficiency, severe loss of bioactivity, complicated operation procedures, and prominent batch-to-batch variation [63].

In recent years, the integrated in situ functionalization fabrication technology for MCs has been developed. Based on high-efficiency reaction systems including photo–click chemistry, in situ copolymerization, coordination bonding, and biotin–avidin specific binding, this technology enables precise and homogeneous incorporation of functional molecules (such as cell-adhesive peptides, growth factors, anti-inflammatory cytokines, targeting moieties, and stimuli-responsive moieties) into the surface or polymer skeleton of microcarriers synchronously with the molding process, realizing a one-step fabrication strategy featured by “functionalization upon molding”.

**Figure 6:**
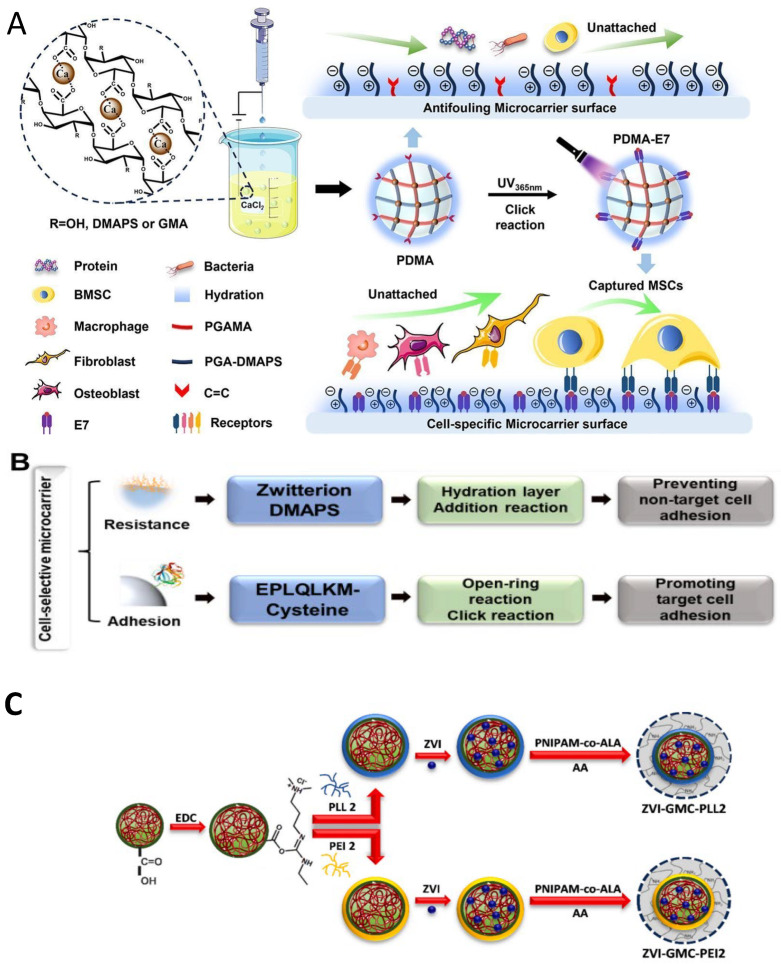
(**A**) Schematic diagram of cell-specific microcarrier preparation process and the principle of specific binding between BMSC and microcarrier. (**B**) Description of the cell-specific microcarrier preparation process. (Reproduced with permission. [64] 2024, Elsevier). (**C**) GMC pre-coated with PEI or PLL, followed by ZVI NP anchoring and PNIPAM-co-ALA grafting. Ascorbic acid (AA), 1-Ethyl-3-(3-dimethylaminopropyl)-carbodiimide hydrochloride (EDC), polyethyleneimine (PEI), polylysine (PLL), zero-valent iron (ZVI). (Reproduced with permission. [65] 2026, Elsevier).

A cell-specific microcarrier (CSMC) with excellent biocompatibility, low background noise, and high-precision was developed, which enables efficient shielding of non-target cells and specific capture of MSCs (Figure 6A,B). The CSMC was fabricated by grafting zwitterionic monomer [2-(methacryloyloxy) ethyl] dimethyl-(3-sulfopropyl) ammonium hydroxide (SBMA) and glycidyl methacrylate (GMA) onto a polygalacturonic acid (PGA) backbone. SBMA forms a hydration layer via solvation to provide excellent antifouling performance, while GMA offers active sites for thiol–epoxy click reaction with sulfhydryl-modified E7 peptide to achieve rapid peptide immobilization. The CSMC showed robust anti-adhesion property against non-specific cells, and the introduction of E7 peptide precisely balanced the hydration layer shielding effect, supporting normal adhesion and proliferation of target BMSCs. This simple, mild, rapid, and widely applicable preparation strategy provides a flexible design platform for bioactive molecule immobilization, and the resultant CSMC also offers guidance for the design of microcarriers with specific biofunctionalization [64].

A thermoresponsive, magnetically responsive microcarrier platform (ZVI-GMC) was prepared through the hybridization of macromolecules and nanoparticles (Figure 6C). The microcarrier consists of a gelatin core functionalized with poly(N-isopropylacrylamide)-allylamine (PNIPAm-ALA) and embedded zero-valent iron nanoparticles (ZVI NPs). The ZVI-GMC system established a robust and biofunctional microcarrier platform for the expansion and differentiation of ADSCs. The results indicated that ZVI-GMC enables enzyme-free, temperature-mediated cell harvesting, with well-maintained cell viability, stemness markers expression (CD90, CD105, CD73), and multipotency. Meanwhile, ZVI NPs incorporation not only improves the structural/thermal stability of microcarriers, but also boosts the proliferative and metabolic activity of ADSCs as confirmed by CCK-8 assay [65].

This fabrication technology not only greatly simplifies the preparation process of microcarriers and improves batch consistency, but also realizes the multi-dimensional functional integration of microcarriers. It can simultaneously satisfy multiple requirements including high-density expansion of stem cells, precise regulation of directed differentiation, and real-time monitoring of therapeutic efficacy, thus serving as the core research direction for intelligent microcarriers in the future.

These advanced MC fabrication technologies enable precise regulation of the structure and properties of MCs, and have respective advantages and disadvantages in SC culture, scalable production, and other aspects, as detailed in Table 2. In fact, each established fabrication method carries well-recognized trade-offs that have yet to be fully resolved. Microfluidic fabrication delivers excellent monodispersity, but it is constrained by inherently low throughput and elevated production costs. Electrospraying, by contrast, is readily scalable, though multi-needle array setups are prone to electric field crosstalk that erodes batch-to-batch consistency. To narrow the gap between particle quality and manufacturing scalability, one viable approach is to pair microfluidic pre-focusing modules with electrospray hardware, preserving strong monodispersity while lifting overall output. For multi-needle systems specifically, targeted electric field shielding and refined parameter calibration can further suppress crosstalk and improve product uniformity across production runs. In addition, as an emerging technology, integrated in situ functionalization fabrication can achieve the simultaneous integration of MC preparation and functionalization, providing a direction for the development of intelligent MCs. The optimization and upgrading of various technologies, as well as cross-technology integration, may be the key to breaking through bottlenecks and promoting the clinical translation of MCs.

## 5. Programmable Microcarrier-Directed Stem Cell Fate Regulation

**Figure 7:**
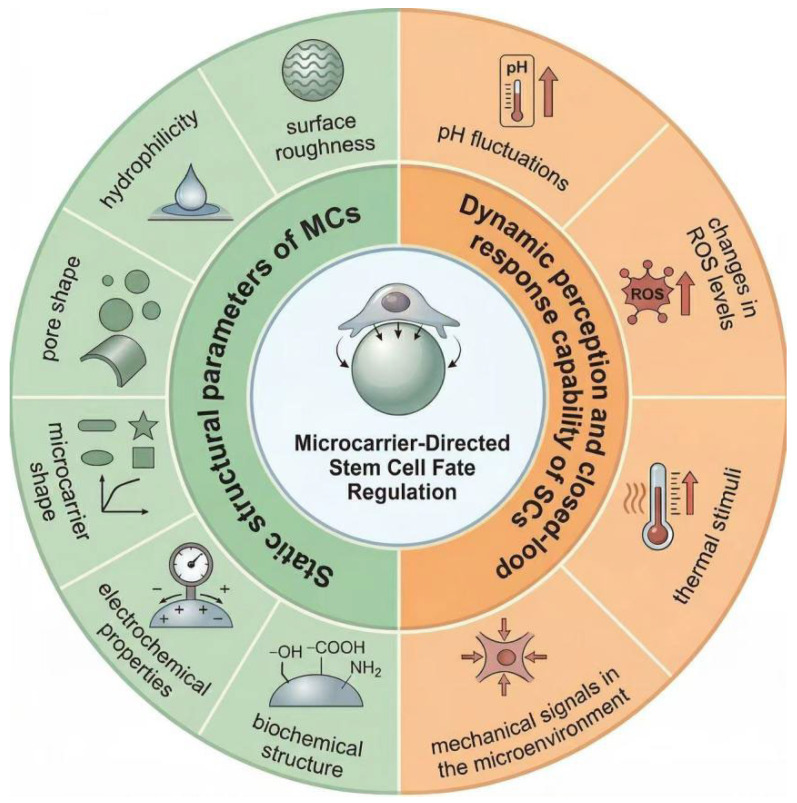
Programmable microcarrier-directed stem cell fate regulation. The static structural parameters of MCs include pore size, curvature, surface roughness, and microcarrier shape, in synergy with their 3D structural features such as stiff, hydrophilicity, electrochemical properties and biochemical structure. The dynamic intelligent regulatory capability of microcarriers lies in the dynamic recognition of pH fluctuations, changes in reactive oxygen species (ROS) levels, thermal stimuli, and mechanical signals in the microenvironment during the in vitro culture or in vivo tissue repair of SCs. Created by Microsoft PowerPoint.

The fate determination and regulation of SCs behaviors depend not only on the 3D microenvironment constructed by the static structural parameters of programmable MCs, but also on the dynamic perception and closed-loop response capability of intelligent MCs to in vitro culture or in vivo regeneration microenvironment of SCs. Their synergistic effect enables precise regulation of the whole-life-cycle behaviors and stable stemness maintenance of SCs. The detailed regulatory parameters are illustrated in Figure 7, and each parameter is systematically elaborated in detail in the subsequent sections.

### 5.1. Static Topographical and Architectural Cues in Regulating Stem Cell Behaviors and Stemness Maintenance

The static structural parameters of MCs, including pore size, curvature, surface roughness, and microcarrier shape, in synergy with their 3D structural features such as stiff, hydrophilicity, electrochemical properties and biochemical structure, provide a favorable microenvironment and high specific surface area, modulating cell-MC interactions and thereby regulating SC behaviors (including adhesion, proliferation, migration, differentiation, etc.) and stemness.

#### 5.1.1. Pore Size of Microcarriers

The pore size of microcarriers is a critical structural parameter that determines the performance of stem cell culture, with its regulatory effects spanning the entire process of cell growth, proliferation, differentiation, and large-scale production. Chen et al. has successfully prepared gelatin methacrylamide porous microcarriers (GelMA PMS) with adjustable pore sizes (10–45 μm) through ice-templating combined with freeze-drying technology, based on the regulation of different freezing temperatures. Among them, PMS 60 with pore size of 25 μm effectively supported uniform cell spreading on its surface, exhibiting the highest cell attachment rate (up to 90.2%) and a spreading area of 0.516 mm^2^/cell, which significantly improves the in vitro expansion efficiency of BMSCs and effectively maintains their stemness characteristics. The results confirm that an appropriate pore size better simulated the SC niche in the natural microenvironment, providing favorable support for BMSCs adhesion, spreading, proliferation, and stemness maintenance [66].

Based on pore size, microcarriers can be classified into three major categories: smooth, microporous, and macroporous microcarriers, each with its benefits and challenges. Generally, cells predominantly adhere to the outer surface of smooth and microporous microcarriers. Smooth microcarriers are ideal for cells not affected by shear [67]. Microporous microcarriers have small pores on the surface, which provides increased surface area for better cell anchorage. In contrast, macroporous microcarriers feature an interconnected internal pore network with ample spatial accommodation, enabling cells to infiltrate, adhere, and grow within the interior of the microcarriers [68]. However, the regulatory effect of microcarrier pore size on stem cell fate is influenced by multiple factors, including the intrinsic properties of the microcarriers and stem cell types. Collagen-modified chitosan porous microspheres (ColMS) with tunable pore sizes (6–18 μm) support adipose-derived stem cell (ADSC) adhesion, proliferation, and enhanced cell viability during injection, offering a promising clinical strategy for the treatment of corneal alkali burns [69]. Microporous annealed particle (MAP) microgel diameter can be tuned to control scaffold pore size (3107, 4637, and 7547 µm^3^) and, in turn, modulate MSC survivability, proliferation, metabolism, and migration, thereby enhancing bioactivity and guiding future applications of MAP for regenerative medicine [70]. Conversely, an excessively small pore size (<10 μm) of microcarriers significantly restricts the migration and infiltration of MSCs into the interior of the microcarriers, while an overly large pore size (>50 μm) may reduce MSCs adhesion efficiency and induce hypoxia in the inner core region of the microcarriers [71].

**Figure 8:**
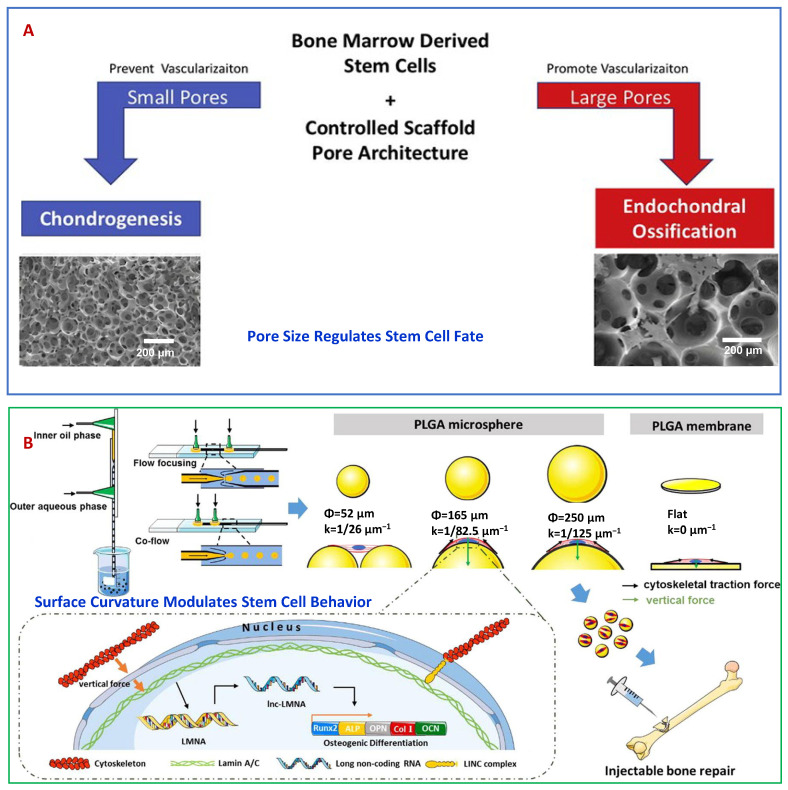
(**A**) Pore size (reproduced with permission. [72] 2020, Elsevier) and (**B**) curvature of microcarriers regulating stem cell fate and function (reproduced with permission. [74] 2021, Wiley).

The long-term stable maintenance of stem cell is a prerequisite for cell therapy, while the pore size of microcarriers exerts a marked regulatory effect on the stemness maintenance of stem cells. Its mechanism is that pore size modulates cell morphology and cytoskeletal tension, and further regulates stemness-related signaling pathways via cellular mechanotransduction. At the regulatory level of stem cell differentiation, the 3D architecture of macroporous microcarriers can induce the directed differentiation of stem cells toward specific lineages, as exemplified by promoting the neuronal differentiation of neural progenitor cells (NPCs) and supporting the multi-lineage differentiation of MSCs, including osteogenic and chondrogenic differentiation (Figure 8A). For neural lineage cell culture, heparin-based microcarriers show marked advantages over traditional 2D culture: they establish a pro-neurogenic niche that supports high-efficiency neuronal differentiation (22% MAP2-positive cells), and simultaneously achieve sustained long-term expansion and superior differentiation performance of NPCs [72]. Peter X Ma et al. reported that small-pore (125–250 μm) poly (l-lactic acid) microcarriers support enhanced chondrogenic differentiation and cartilage formation compared to large-pore (400–625 μm) scaffolds. Endochondral ossification is prevented in microcarriers with very small pore sizes (60–125 μm). The results indicated that pore size is a critical microcarrier design parameter to induce multiple BMSC differentiation fates [73].

#### 5.1.2. Curvature of Microcarriers

Microcarrier curvature regulated the cell migration, proliferation/differentiation as well as the underlying signaling pathway of stem cells (Figure 8B) [74] Low-curvature microcarrier surfaces promote stem cell spreading and growth. High-curvature surfaces, by contrast, steer stem cells toward bone or cartilage development through controlled cell skeleton changes and mechanical signal responses, alongside modified cell attachment [75,76]. Werner et al. [77] constructed a resin microchip with biomimetic 3D-curved surfaces to recapitulate the curvature characteristics of microcarriers, and found that increased convex curvature outperformed flat and concave surfaces in enhancing osteocalcin levels of hMSCs via tuning cytoskeletal forces exerted on the nucleus. They further validated that surface curvature variation acts as a key biophysical regulator to induce cytoskeletal remodeling, promote myosin-generated contractility [78] and modulate the RhoA/ROCK signaling pathway, thus governing the functional behaviors of hMSCs [79]. Mono-distributed PLGA microspheres of curvatures (κ) from 1/26 µm^−1^ to 1/125 µm^−1^ were produced with a microfluidic device to regulate BMSCs osteogenic differentiation potential. The PLGA microcarriers of κ = 1/82.5 µm^−1^ was shown to provide the most suitable microenvironment for BMSCs to grow and undergo osteogenic differentiation. In addition, the PLGA microcarriers of κ = 1/82.5 µm^−1^ was found to significantly enhance the F-actin cytoskeletal organization, nuclear distortion and expression of Lamin A [80].

#### 5.1.3. Stiff

Stem cells sense the matrix stiffness and respond differently with their shapes and fates. Matrix stiffness significantly influences stem cell behavior and function. Three microcarriers with gradient stiffnesses (soft: 0.15 MPa, medium: 1.86 MPa, stiff: 8.57 MPa) were fabricated to mimic amniotic membrane (AM), commercial microcarriers and sclerotic tissue, respectively, and stiffened tissue conditions. The results showed that soft-matrix microcarriers with AM-like stiffnesses (~0.15 Mpa) significantly enhanced cell proliferation. Furthermore, human amniotic epithelial cells (hAECs) cultured on the soft-matrix microcarriers exhibited upregulated expression Wnt proteins (Wnt7A, Wnt11), frizzled receptors, and downstream target genes. The result indicated that adjusting microcarrier stiffness to adopt the soft-matrix microcarriers enables efficient production of high-quality and high-yield hAECs [81].

In addition, the proliferation levels of various stem cell types are differently influenced by matrix stiffness. For mesenchymal stem cells (MSCs), rigid surfaces (stiffness of 34 kPa) induce a spindle-like morphology and drive osteogenic differentiation, while softer substrates (i.e., 1 kPa) preferentially promote chondrogenic, adipogenic or neuronal differentiation. Substrates with intermediate stiffness are optimal for myogenic lineage specification. Stiffness-dependent proliferation patterns also vary widely across stem cell types from different tissue sources. The maximum proliferation capacity of BMSCs occurs on matrices with 2.51 MPa stiffness [82]. In contrast, relatively soft matrices are more favorable for the proliferation of other stem cell populations: the optimal stiffness is 3.52 kPa for adipose-derived MSCs [83], 3.5 kPa for adult neural stem cells (NSCs) [84], and 13–16 kPa for umbilical cord-derived MSCs (UC-MSCs) [85]. For muscle progenitor cells, a moderate stiffness of 48–53 kPa yields the highest proliferation level.

**Figure 9:**
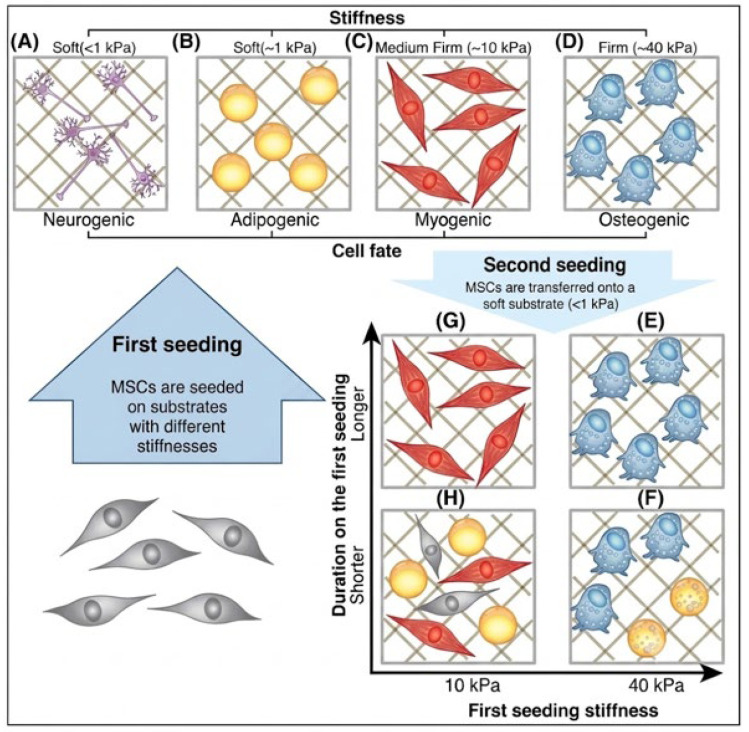
Effects of microcarrier stiffness on the differentiation of MSCs. Variable stiffness of microcarriers during preculture drives the preferential differentiation of MSCs down distinct lineages: neurogenic (**A**), adipogenic (**B**), myogenic (**C**), and osteogenic (**D**). After preculturing on microcarriers with specific stiffness for variable durations and subsequent transfer onto a soft substrate for culture, MSCs differentiate down the osteogenic lineage (**E**), heterogeneous osteogenic/adipogenic lineages (**F**), myogenic lineage (**G**), or heterogeneous myogenic/adipogenic lineages (**H**). Reproduced with permission. [86], 2014, Nature Portfolio. Reproduced with permission. [87], 2013, AAAS. Reproduced with permission. [88], 2017, BioMed Central.

After preculturing on microcarriers with variable stiffness, MSCs undergo preferential differentiation down specific cell lineages [86,87,88] (Figure 9).

It has been demonstrated that preculturing on soft microcarriers suppresses the in vivo fibrogenesis of MSCs [18,89], while the in vivo engraftment efficiency of stem cells is directly correlated with the elasticity of the microcarriers used for in vitro expansion [90,91]. Taken together, the mechanical memory effect of MSCs is a key consideration for the development of tailored, MSC-compatible microcarriers.

#### 5.1.4. Electrochemical Properties

Exchange capacity and charge density are the two electrochemical properties of the positively charged microcarriers which have been evaluated regarding the cell attachment and proliferation. Generally, charge density reflects the capability of a substratum for ion exchange, while exchange capacity of a charged surface is an intrinsic parameter which is measured when the attached molecules to a polymer backbone are fully protonated at low pH (pH < 2). It has been found that a minimum value of exchange capacity is required to support cell attachment and growth onto the microcarriers [92]. The minimum value is dependent on the type of the substratum. For instance, no growth was observed for the BHK, MDCK, CEF, and MRC-5 cells under the exchange capacity of 0.58 meq/g dry primary amino derivatized polyacrylamide (PAA) beads, while this threshold was 1.8–2 meq/g for dry tertiary amino-derivatized PAA beads [93]. By increasing the exchange capacity of the substratum from this minimum value, cell attachment and growth was improved. Based on the type of the amino group-derivatized substratum, the rate of this enhancement would be sharp or gradual. However, there is an upper threshold above which cell attachment decreases [94] or does not change significantly. Regarding charge density, it has been reported that there is no direct correlation between charge density and cell attachment ratio as observed for CHO cells on DEAE-HCL-derivatized dextran beads [95].

#### 5.1.5. Microcarrier Shape

The shape characteristics of microcarriers have been widely reported to exert a regulatory effect on stem cell behaviors. Commonly microcarriers are available with spherical, cylindrical lens and hexagon shapes. Driven by the inherent mechanosensitivity of stem cells to mechanical cues and biophysical properties of culture substrates, the diverse shapes of microcarriers exert distinct regulatory effects on stem cell proliferation and differentiation, thus requiring targeted design and selection of microcarriers for specific culture outcomes. Spherical microcarriers with a diameter of 100–300 mm remains the most common shape in stem cell culture [4]. Given their large cell size and adherent monolayer growth phenotype, MSCs need microcarrier spheres of ~150 mm in diameter to enable sufficient cell adhesion and spreading. Conversely, human pluripotent stem cells (hPSCs) exhibit a native multicellular aggregate growth mode, and thus favor microcarrier spheres smaller than 100 mm in diameter to promote the spontaneous co-aggregation of cells and microcarriers into compact clusters [96].

#### 5.1.6. Surface Roughness

The regulation of surface roughness on stem cell behaviors presents a significant scale-dependent effect: macro-scale roughness (100 μm–1 mm) can provide sufficient spatial support for the attachment and spreading of large-volume cell clusters, and improve cell viability in large-scale culture systems [97,98]; micro-scale roughness (100 nm–100 μm) can greatly increase cell adhesion sites, promote ordered cytoskeleton arrangement and cell cycle progression, significantly improve the expansion efficiency of stem cells, and inhibit spontaneous differentiation of cells [99]; nanoscale roughness (<100 nm) can effectively maintain the stable expression of core stemness markers during long-term subculture, reduce the loss of stem cell stemness, while maintaining high cell viability and proliferation potential by activating signaling pathways related to cell–matrix interactions [100,101]. The above-mentioned surface roughness can be precisely regulated by acid etching, plasma treatment, alkali treatment, and nanonization modification.

#### 5.1.7. Hydrophilicity

Surface hydrophilicity affects the adhesion behavior and expansion potential of SCs. A moderately high hydrophilic microcarrier surface can optimize the adsorption amount and native conformation of extracellular matrix proteins such as fibronectin and laminin, enhance the stable formation of focal adhesions, which not only significantly improves the adhesion efficiency and proliferation rate of SCs, but also reduces the apoptosis level and maintains high cell viability during in vitro expansion [102]. Meanwhile, a suitable hydrophilic environment can accurately simulate the physicochemical characteristics of SC niche in vivo, inhibit non-specific spontaneous differentiation of SCs, and effectively maintain their multi-directional differentiation potential and stemness phenotype during long-term in vitro expansion. In contrast, an excessively hydrophobic surface tends to cause abnormal conformation of adsorbed proteins, resulting in poor cell adhesion, decreased expansion efficiency and loss of stemness.

#### 5.1.8. Biochemical Structure

The biochemical structure of the microcarrier surface, including functional group types, charge characteristics and biostructural domains, can precisely regulate the biological behaviors of SCs at the molecular level. Surface functional groups (such as -OH, -NH_2_ and -COOH groups) can regulate the physicochemical properties of the surface microenvironment to affect intracellular signal transduction, thereby modulating the proliferation activity and stemness maintenance of SCs. It has been shown that -NH_2_ groups promote MSC proliferation, spreading and osteogenic commitment, while lowered MSC spreading and enhanced chondrogenesis has been observed on the surfaces with increased concentration of -COOH groups [103].

Precise regulation of surface charge can optimize cell behaviors without exogenous biological factors: a moderately negatively charged or weakly positively charged surface can promote cell cycle progression and increase the expansion fold, while excessive positive charge is prone to induce cytotoxicity and disrupt stemness homeostasis.

Modification of specific biostructural domains such as the RGD sequence can precisely balance the proliferation, differentiation and stemness maintenance of SCs through the integrin-mediated signaling pathway. It can not only significantly improve the in vitro expansion efficiency and cell viability of stem cells, but also stably maintain their multi-directional differentiation potential during long-term culture [104].

### 5.2. Dynamic Regulatory Properties of Intelligent Microcarriers on Stem Cells: Sensing and Response

The dynamic intelligent regulatory capability of microcarriers lies in the dynamic recognition of pH fluctuations, changes in reactive oxygen species (ROS) levels, thermal stimuli, and mechanical signals in the microenvironment of SCs. Based on the above signal recognition, microcarriers can achieve spatiotemporally controlled, real-time adjustment of their physicochemical properties, release behavior of bioactive molecules, and surface topological structure, which in turn precisely meet the dynamic physiological requirements of SCs.

#### 5.2.1. Magneto-Stimulation Response

**Figure 10:**
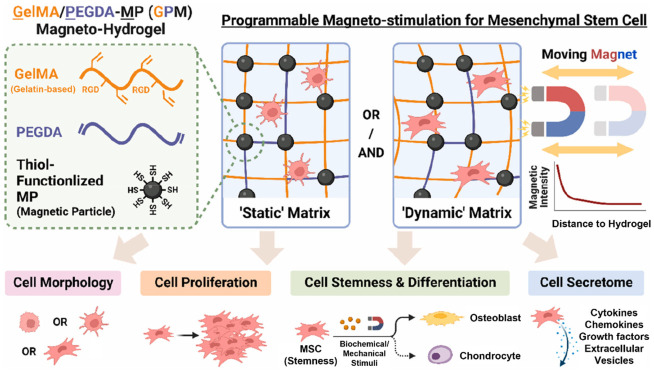
Schematic illustration of GelMA/PEGDA-MP (GPM) magnetic hydrogel microcarriers to provide dynamic mechanical stimulation to influence MSC morphology, proliferation, stemness, differentiation, and secretome profile. Reproduced with permission. [107] 2023, Elsevier.

MSCs can sense mechanical signals from their microenvironment through cell surface receptors like integrins and then transduced the signals to cytoskeleton and nucleus, to accelerate proliferation, and boost growth factor production [105,106]. Magnetic forces facilitate dynamic cell culture by employing magnetic fields to exert mechanical stress and stimuli. A magneto-responsive 3D dynamic hydrogel (GPM hydrogel) that harnesses magnetically induced dynamic mechanical stimulation (DMS) to enable the integrated manufacturing and therapeutic application of MSCs (Figure 10). 

This hydrogel enhances MSC proliferation with negligible impairment of stemness, thus improving the quantity and quality of MSC. Mechanistically, magneto-actuated DMS strengthens matrix-integrin β1 interactions to promote MSC spreading and proliferation. Meanwhile, it modulates the MSCs by activating key mechanotransduction cascades, including FAK-ERK pathway activation and YAP nuclear translocation. Specifically, DMS can direct the differentiation of MSCs and boost their paracrine activity. More importantly, this all-in-one platform combines the whole complex process into a single system from MSC preparation to clinical application [107].

For another instance, a sustainable alginate-based magneto-responsive microcarrier (CP/Fe_3_O_4_/SA) with autonomous oxygen-generating function was developed for bone marrow stromal cell (BMSC) culture. The microcarrier employs polylactic acid microsphere-encapsulated calcium peroxide (CP) as an endogenous oxygen reservoir, and exhibits excellent responsiveness to external magnetic fields. Under magnetic field stimulation, BMSCs seeded on the microcarriers presented significantly enhanced viability, markedly downregulated hypoxia-inducible factor-1α (HIF-1α) expression, and upregulated osteogenic gene expression [108].

Magneto-responsive microcarriers can remotely and non-invasively penetrate biological barriers, target stem cells, and effectively regulate their adhesion, proliferation and paracrine functions. This microcarriers boost stem cell survival and target in vivo differentiation, while avoiding potential safety risks from gene editing and cytokine use. Future research will focus on developing highly biocompatible, tunable magneto-sensitive microcarriers, and investigating the system’s in vivo targeted delivery efficiency and clinical application potential.

#### 5.2.2. pH Response

pH-responsive MCs are fabricated from pH-sensitive polymers including polyhistidine-modified GelMA, poly(β-amino ester) (PBAE) block copolymers, and 2-(dimethylamino)ethyl methacrylate (DMAEMA)-grafted gelatin. Nuran Işıklan developed pH-responsive chitosan-coated pectin-graft-poly(N,N-diethyl acrylamide) (Pec-g-PDEAAm/CS) microcarriers containing 5-Fluorouracil (5-FU) as a model drug [109].

Novel core–shell pH-responsive metal–organic framework (MOF) microcarriers with polyelectrolyte hydrogel coatings were fabricated via layer-by-layer self-assembly. Electrostatic interactions among ZIF-8-NH_2_, polystyrene sulfonic acid (PSS) and cationic gallic acid-grafted chitosan (CS-g-GA) stabilized the multilayer network, forming a robust hydrophilic hydrogel shell. This shell improved the microcarriers’ wettability, colloidal stability, cytocompatibility, and iodine-loading capacity. The I_2_@ZIF-8-NH_2_@Gel microcarriers showed pH-responsive iodine release, functioning as a smart “on-off” switch for targeted antimicrobial delivery. Notably, selective, sustained iodine release was triggered exclusively at pH < 5.5, matching the acidic microenvironment of bacterial infections. This pH-triggered on-demand release enables targeted iodine delivery to infected sites, ensuring efficient release at the pathological microenvironment and excellent bactericidal activity [110].

pH is a stimulus to facilitate detachment of stem cells from microcarriers or their release for in situ tissue engineering applications. However, the activity of cell metabolism-controlling enzymes is strongly pH-dependent, and thus using pH reponse can affect the growth and differentiation of stem cells. It has been reported that suboptimal pH can lead to inhibition or altered differentiation of stem cells. As such, the limited pH range of 6.8–7.4 for normal cell functions is the main reason of the few studies on pH stimulus to produce microcarriers [111].

#### 5.2.3. Thermoresponsiveness

In large-scale stem cell expansion systems, efficient and gentle cell harvest is the key factor that determines expansion efficiency, cell viability, and functional integrity. Protease digestion is the most used method for cell harvest. However, it has limited separation efficiency, and will irreversibly damage cell membrane surface proteins and degrade the ECM. This eventually leads to reduced cell viability, impaired stemness, and decreased differentiation potential.

Poly(N-isopropylacrylamide, PNIPAM) is a widely used thermoresponsive polymer. Its characteristic lower critical solution temperature (LCST) is around 32 °C, and it has a reversible thermally induced phase transition property. When the temperature is above the LCST, PNIPAM molecular chains coil into a globular structure with a hydrophobic surface. This surface can adsorb ECM proteins to promote cell adhesion. When the temperature drops below the LCST, the molecular chains stretch into a random coil conformation, and surface hydrophilicity increases sharply [112]. This allows cells to detach completely along with their secreted ECM, avoiding the inherent drawbacks of traditional enzyme digestion and meeting the needs of gentle stem cell harvest.

Chen et al. developed thermoresponsive PNIPAM/GelMA composite hydrogel microcarriers with uniform particle size and excellent dispersibility, for 3D expansion and enzyme-free harvest of BMSCs. Thermoresponsive PNIPAM was used as the functional component for enzyme-free cell harvest, and gelatin methacryloyl (GelMA) as the functional component to support cell adhesion and expansion. The microcarriers were fabricated via microfluidic synchronous photo crosslinking combined with ice template freeze-drying method. The results showed that the composite microcarriers had excellent thermoresponsive performance. They supported stable proliferation of BMSCs for 7 days. Efficient detachment of BMSCs from the microcarrier surface was achieved within 1 h at room temperature. No obvious cell viability loss was observed during the entire harvest process, and the detached cells still maintained stable proliferation capacity for another 7 days [66].

It is worth noting that PNIPAM segments undergo volume shrinkage during the thermally induced phase transition. This easily generates shear stress on cells attached to the microcarrier surface, leading to cell damage and decreased cell viability. Therefore, the key future research direction for this type of thermoresponsive microcarriers is to effectively suppress volume change during phase transition and reduce the adverse effects of shear stress on cells, while retaining the excellent thermoresponsive detachment performance of PNIPAM/GelMA composite microcarriers.

#### 5.2.4. ROS-Responsive Properties

ROS levels in stem cells vary significantly between quiescent, proliferative, and differentiated states. Accumulating evidence demonstrates that ROS act not only as metabolic byproducts, but also as pivotal signaling regulators of stem cell fate and functional homeostasis. ROS levels are dynamically regulated, and bidirectionally and reversibly shape the function and lineage bias of distinct stem cell. Specifically, ROS-high short-term repopulating stem cells show enhanced hematopoietic reconstitution and a prominent myeloid lineage bias, while ROS-low quiescent long-term repopulating stem cells sustain robust long-term self-renewal [113].

**Figure 11:**
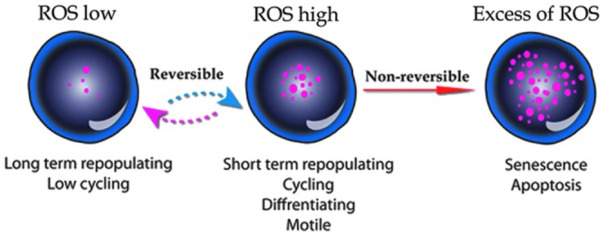
Intracellular reactive oxygen species (ROS) regulate self-renewal versus activation of hematopoietic stem cells. Reproduced with permission. [114] 2023, Elsevier.

Physiological ROS concentrations are essential for preserving stem cell self-renewal potential. In the tissue niche, stress- and inflammation-driven aberrant ROS upregulation induces lineage-specific stem cell differentiation, whereas chronically insufficient ROS levels cause stem cell dysfunction (Figure 11). Thus, developing a microcarrier system that enables precise, dynamic tuning of intracellular ROS levels is critical for maintaining endogenous stem cell homeostasis and protecting immune barrier integrity.

The key role of reactive oxygen species (ROS) in intervertebral disk degeneration (IVDD) pathogenesis makes antioxidant therapy a promising IVDD treatment. Zhang et al. constructed ROS-responsive magnesium-containing microspheres (Mg@PLPE MSs) with a core–shell structure comprising poly(lactic-co-glycolic acid) (PLGA) and ROS-responsive polymer poly(PBT-co-EGDM) as the shell, and magnesium microparticles as the core. The Mg@PLPE MSs can effectively scavenge overproduced ROS by simultaneously reacting with H_2_O_2_ and •OH, thus inhibiting ECM degradation. As a result, IVDD rats treated with Mg@PLPE MSs exhibit minimal nucleus pulposus loss, less ECM degradation, minimal radial fissures of the annulus fibrosus, and a higher disk height index [114].

In addition to regulating ROS concentrations, functional modification of microcarriers represents another important strategy for achieving protective stem cell delivery. For example, introducing ROS-sensitive groups, such as thioketals, selenoethers, and boronic esters, onto the microcarrier surface enables them to respond to elevated ROS levels in the pathological microenvironment, thereby achieving targeted drug release or protective stem cell delivery.

The role of ROS in regulating stem cell fate is well established. Further investigation is warranted to explore how redox signaling in these stem cells can be translated into novel strategies to preserve systemic and local homeostasis, as well as more effective therapies for diabetic cardiovascular complications.

In summary, the synergistic effect of MC static structural parameters and dynamic perception-closed-loop response characteristics can achieve regulation of SC behaviors and long-term stemness maintenance. However, existing MCs still face bottlenecks: non-damaging stem cell consistent retrieval from microcarriers remains a key unmet challenge; the synergistic regulatory mechanism of multi-stimulus responsive MCs remains unclear, and the clinical translation of MCs is limited by issues such as batch-to-batch consistency, in vivo degradation rate matching, and biosafety evaluation. In-depth research is still needed to promote the clinical application of stem cell technologies.

## 6. Functionalized Microcarrier-Mediated Stem Cell Culture and Therapeutic Applications

**Figure 12:**
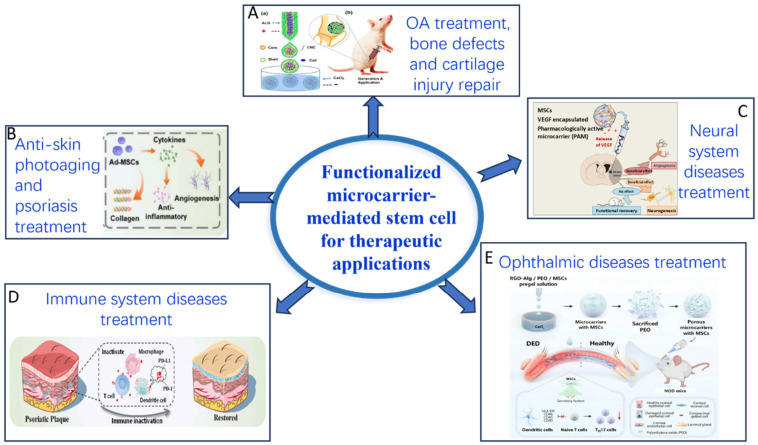
Schematic diagram of microcarrier-mediated stem cell culture system for the treatment of representative relevant diseases. (**A**) OA treatment, bone defects and cartilage injury repair. Reproduced with permission. (**a**) Microcapsules generation process by microfuidic electrospray. (**b**) Stem cell-laden core–shell microcapsules for bone repair. [17] 2022, Springer Ltd. (**B**) Anti-skin photoaging and psoriasis treatment. Reproduced with permission. [18] 2025, Elsevier. (**C**) Neural system disease treatment. Reproduced with permission. [21] 2015, Elsevier. (**D**) Immune system diseases treatment. Reproduced with permission. [19] 2023, Elsevier. (**E**) Ophthalmic diseases treatment. Reproduced with permission. [20] 2025, AAAS.

Microcarriers play pivotal roles in SC therapy [40]. First, through their ultra-high specific surface area and 3D structure, they enable efficient and scalable expansion of SCs while synchronously maintaining SC stemness and intrinsic biological functions, thus providing stable, controllable, and high-quality SCs for clinical applications. Second, they serve as a biomimetic delivery scaffold for SCs, which isolates transplanted cells from damage induced by the in vivo microenvironment, and markedly improves the in vivo retention rate and survival rate of SCs. In addition, SCs have been reported to have various therapeutic functions, and thus have been actively tested for their therapeutic potential against a wide range of diseases [115]. At present, the functionalized MC-mediated SC therapeutic system has achieved breakthrough advances. Herein, we systematically elaborate on the application progress of MC-mediated SCs in the treatment of a wide spectrum of major diseases (Figure 12).

### 6.1. Immune System Diseases Treatment

Autoimmune diseases are clinically intractable. Chronic autoimmune diseases often cause irreversible target organ damage. It was reported that MSCs increase infiltration of peripheral immune cells into CNS and skew the infiltrated immune cells toward regulatory T lymphocytes (Treg) and Th2 lymphocytes. Treg and Th2 secret anti-inflammatory cytokines such as IL-4, IL-10, and TGF-β [116]. MSCs show promise for their immunomodulatory and anti-inflammatory activities, and microcarriers are crucial for scalable expansion of MSCs and enhancement of therapeutic functions.

Umbilical cord-derived MSCs (UC-MSCs) exhibit higher proliferative capacity and require less invasive harvesting procedures than other MSCs sources. Critically, UC-MSCs have immunomodulatory effects comparable to those of BM-MSCs, and this bioactivity is maintained even after 10 passages. For this purpose, the authors employed polystyrene CellBIND surface microcarriers to investigate the harvest efficiency, batch consistency, and cost reduction in UC-MSCs cultured in 500 mL bioreactors. Furthermore, to evaluate the immunomodulatory efficacy of MSCs, the therapeutic effects of both ST-UC-MSCs and MC-UC-MSCs were assessed. The results demonstrated that UC-MSCs produced in a suspension culture system retained their therapeutic efficacy in a mouse graft-versus-host disease (GvHD) model, and multiple doses showed better therapeutic than single infusion. However, further studies are needed to achieve scale-up of bioreactor capacity and reduction in cryopreservation time [117].

**Figure 13:**
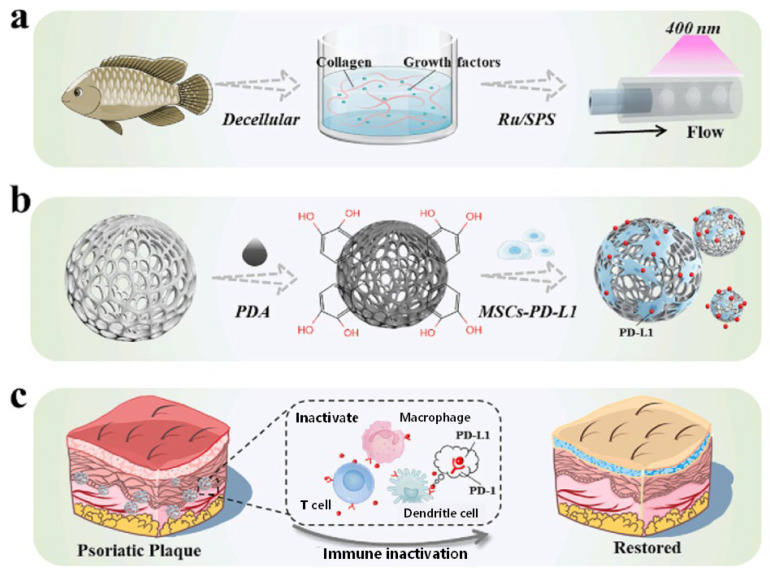
(**a**) Schematic illustration of the decellular process from fish skin for the preparation of the microcarriers; (**b**) schematic image of fabrication of MSCs-loaded microcarriers; (**c**) application of the MSC-loaded microcarriers for the treatment of psoriasis. Reproduced with permission. [19] 2023, Elsevier.

The stem-cell-therapy supported by hydrogel microcarriers has shown potentials in the regulation of autoimmune diseases. Trends in this field focus on enhancing the viability and the delivery controllability of stem cells. Novel fish ECM microcarriers loaded with MSCs that secrete programmed cell death-ligand 1 (PD-L1) for autoimmune disease treatment (Figure 13). With the decoration of polydopamine, MSCs with enhanced PD-L1 expression by lentivirus transfection can adhere well to these ECM microcarriers. MSCs delivered through the ECM microcarriers have a higher survival rate compared with direct injection. In addition, these MSC-loaded microcarriers successfully inhibited the epidermis thickening and lowered the PASI score by decreasing immune cell infiltration. Optimizing stem cell maintenance and immunomodulation ability for different stem cell types on the microcarriers requires further investigation [19].

Collectively, these results validate the superiority of MC-based MSC therapy for immune disorders.

### 6.2. Application in OA Treatment, Bone Defects and Cartilage Injury Repair

Bone and cartilage tissues have poor self-repair ability after injury. Based on MC technology, the stem cell therapy is a promising approach for osteochondral regeneration. Its clinical use is severely limited by poor in vivo retention of transplanted cells, impaired paracrine activity, and the lack of scalable culture systems to produce high-quality SCs. Therefore, the combining stem cell therapy with microcarrier (MC) technology is expected to effectively overcome these limitations.

**Figure 14:**
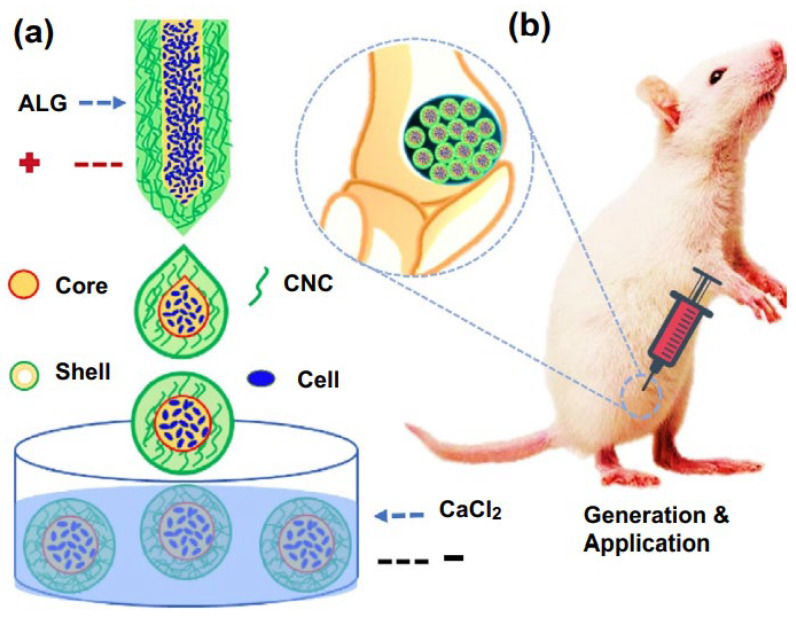
Schematic illustrations of the generation and application of the stem cell-laden microcapsules. (**a**) Microcapsules generation process by microfuidic electrospray. (**b**) Stem cell-laden core–shell microcapsules for bone repair. Reproduced with permission. [17] 2022, Springer Ltd.

For the treatment of bone defects, Yang et al. employed an all-aqueous phase microfluidic electrospray approach to prepare core–shell microcarriers loaded with BMSCs (Figure 14a) [17]. Introducing cellulose nanocrystals into the shell layer roughens the surface of the microcapsules and creates small pores, which enhances the mechanical strength of the microcarriers and promotes cell growth. After injecting the microcarriers into the bone defect sites of SD rats for 8 weeks, the results indicated that the BMSC-laden microcarriers effectively enhanced bone regeneration (Figure 14b), with notable improvements in bone volume fraction and bone mineral density.

To address the bottlenecks of stem cell therapy for osteoarthritis (OA), namely insufficient in vivo retention efficiency and restricted paracrine function, Ren et al. explored the regulatory effects of spherical gelatin microcarriers with varying particle sizes on the biological functions of adipose-derived stem cells (ADSCs), and further evaluated the therapeutic potential of Microcarriers@ADSCs for OA in vivo. The results revealed that gelatin microcarriers significantly boosted the paracrine capacity of ADSCs. Microcarriers@ADSCs effectively improved the lubrication properties of the cartilage interface, and modulated cartilage metabolism in vivo, concurrently markedly suppressing local joint inflammation and reversing the pathological progression of OA. Additionally, this delivery system enabled the low-dose intra-articular administration of ADSCs, with a significant enhancement in the in vivo retention efficiency of the stem cells [118]. This MC-based stem cell therapeutic system takes advantage of its biomimetic 3D structure to support scalable expansion of high-quality SCs, while also synergistically improving the cells’ in vivo retention and paracrine regenerative function. Notably, the SCs used in this system are easily obtained through minimally invasive bone marrow aspiration, with abundant autologous sources available.

For the cartilage repair therapy, MSCs are a leading candidate. The in vitro chondrogenic capacity of MSC-laden, porous biodegradable microcarriers made from lightweight polycaprolactone (LPCL-MCs), as well as their therapeutic potential for in vivo osteochondral defect repair, were systematically characterized. The results confirm that chondrogenically differentiated MSC-LPCL-MCs constructs simultaneously support the large-scale expansion and targeted delivery of MSCs, while efficiently driving cartilage repair in rabbit osteochondral defect models [119]. In addition, biodegradable gelatin microcarriers (MCs) loaded with human cartilage progenitor cells (CPCs) or BMSCs were developed to repair the rabbit knee cartilaginous defect. The results indicated that, at 3 months post-implantation, acellular MCs only filled defects with fibrous cartilage; BMSC-loaded MCs filled defects with boundary cartilage-like tissue, but with an incompletely smooth surface; CPC-loaded MCs achieved nearly full defect repair, with regenerated cartilage matching the smooth appearance and structure of normal cartilage. Its repair efficacy and convenience suggest that the strategy of stem-cell-microcarrier construct has promising clinical application prospects [120].

Taken together, the therapeutic strategy combining microcarriers with stem cells effectively addresses the challenges plaguing conventional stem cell therapy, and has demonstrated excellent therapeutic performance and clinical translation potential in applications including bone defect repair, OA treatment, and cartilage injury repair. Looking ahead, further optimization of the scalable manufacturing processes for stem cell-laden MC constructs is still required to underpin the repair of bone and cartilage injuries.

### 6.3. Anti-Skin Photoaging and Psoriasis Treatment

Long-term exposure to ultraviolet (UV) radiation causes skin photoaging and irreversible damage. Stem cell therapy has shown promise for revitalizing aged skin by secreting anti-inflammatory and antioxidant factors. However, intravenously injected stem cells often have low survival rates, and their unpredictable differentiation in vivo leads to inconsistent treatment outcomes. In addition, a single dose injection only provides temporary effects and fails to achieve long-term anti-aging effects [121].

**Figure 15:**
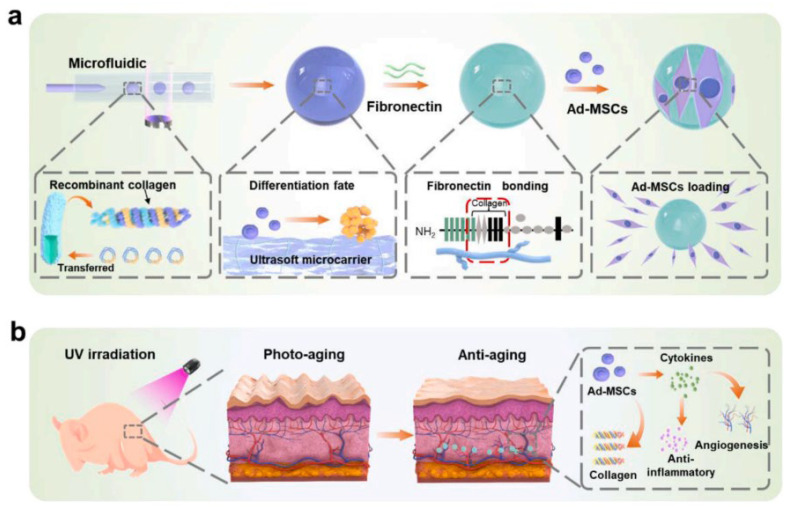
Schematic illustration of (**a**) the construction of the mechanically tunable microcarriers and the cell seeding process, and (**b**) the treatment for photoaging mice using the stem cell-loaded microcarriers. Reproduced with permission. [18] 2025, Elsevier.

MSCs possess strong self-renewal, multi-directional differentiation, and paracrine regulation capabilities. However, the mechanical microenvironment at the cell delivery site is the key factor affecting SC fate, survival efficiency, and therapeutic effect. Zhang et al. prepared mechanically adjustable human recombinant collagen (RHC) microcarriers coated with fibronectin (Fn) by droplet microfluidic technology, and further integrated with the platelet-derived growth factor-BB (PDGF-BB) to efficiently load adipose-derived mesenchymal stem cells (Ad-MSCs) (Figure 15). The results indicated that subcutaneous injection of Ad-MSCs-loaded RHC microcarriers significantly reduced UV-induced skin wrinkles, effectively upregulated collagen synthesis, and increased vascular density [18]. The core role of the mechanically regulated RHC MCs lies in the precise regulation of cross-linking agent concentration and reaction time, which allows adjusting their mechanical properties to the optimal range for SC differentiation. This optimization maximizes SC loading efficiency and therapeutic activity, achieving targeted treatment of photoaged skin. These encouraging results indicate that the mechanically regulated microcarriers have great potential to deliver stem cells and regulate their differentiation for anti-photoaging treatments.

Psoriasis is a chronic inflammatory skin disease limited by treatment relapse, drug resistance, and adverse effects. MSCs are multipotent cells with robust immunomodulatory properties. Chen et al. demonstrated that 3D cultures with recombinant collagen microcarriers markedly enhanced the stemness of human umbilical cord (hUC-MSCs), including pluripotency, immunomodulatory factors secretion, and mesodermal differentiation potential. Mechanistically, the effect of MSCs on murine psoriasis is mediated via the IL-17/NF-κB signaling pathway, and 3D-cultured hUC-MSCs exert an inhibitory effect on skin lesion inflammation than 2D-cultured counterparts. The advantage of 3D-cultured hUC-MSCs is their capacity to mitigate inflammatory responses in psoriatic lesions by reducing immune cell infiltration and regulating the expression of inflammatory cytokines and chemokines. The collagen microcarrier-based 3D culture is an effective method for preparing high-quality MSCs and treating autoimmune disease [122].

### 6.4. Ophthalmic Diseases Treatment

The ocular structure is sophisticated and has limited regenerative capacity. In many ophthalmic diseases, irreversible damage to functional cells means traditional treatments often fail to repair tissues or restore vision, creating a major bottleneck in ophthalmic care. Stem cells are the core seed cells for repairing ocular damage, while microcarriers provide a growth environment that mimics in vivo conditions, solving key challenges in stem cell transplantation. The integrated microcarrier–stem cell system has become a key research focus and promising direction in regenerative ophthalmology.

**Figure 16:**
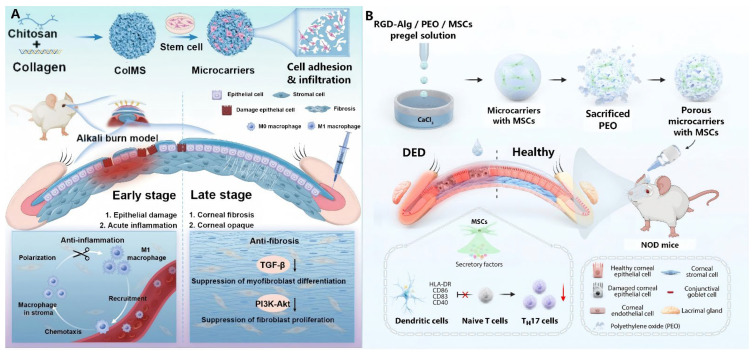
(**A**) Schematic illustration of ColMS-ADSCs preparation and application in corneal alkali burn treatment and functional vision restoration. Reproduced with permission. [64] 2025, Elsevier. (**B**) Schematic of the fabrication of the porous RGD-Alg microcarriers with MSC encapsulation and their application in promoting autoimmune DED in NOD mice. Reproduced with permission. [20] 2025, AAAS.

Corneal alkali burn is a devastating ocular injury that frequently causes irreversible vision loss. Conventional cell injection is hampered by poor cell viability, low lesion retention, and mechanical trauma [123]. A porous microcarrier system (ColMS-ADSCs) of collagen-modified chitosan microspheres laden with adipose-derived stem cells, which exhibits robust immunomodulatory capacity and corneal epithelial regenerative potential. In a rat corneal alkali burn model, subconjunctival injection of ColMS-ADSCs suppressed acute inflammation in the early injury phase, effectively attenuated corneal fibrosis in the late phase, and ultimately markedly restored corneal transparency (Figure 16A). This method drove corneal epithelial regeneration, restoring epithelial thickness to near-normal levels and reducing myofibroblast activation. Specifically, the fluorescence intensity of α-smooth muscle actin, the canonical myofibroblast marker, was 1.5-fold lower in the treatment group than in the control group. Taken together, ColMS-ADSCs provide a promising new avenue for corneal alkali burn treatment and functional vision restoration [64].

Dry eye disease (DED) is characterized by chronic inflammation and an unstable tear film. Although stem cells have shown potential for DED treatment, the major issue is low cell delivery efficiency. Porous arginine–glycine–aspartic acid–modified alginate microcarriers with mesenchymal stem/stromal cells (RGD-Alg@MSCs) were developed as eye drops for autoimmune DED treatment (Figure 16B). These microcarriers enable large-scale cell expansion while maintaining stemness for ocular application. RGD-Alg@MSCs significantly improved cell survival rate, reduced apoptosis and reactive oxygen species, and enhanced release of immunomodulatory factors. In the mouse model, RGD-Alg@MSCs exhibited prolonged ocular retention and enhanced tear production, promoted corneal healing, and suppressed inflammation. This study indicated that RGD-Alg@MSCs substantially improves stem cell delivery efficiency for treating autoimmune DED [20].

The synergistic advantages of the microcarrier–stem cell system offer new ways to treat ophthalmic diseases, effectively repairing damaged ocular tissues and bringing hope to patients unresponsive to traditional therapies. While the technology has made preliminary progress, it still faces challenges such as material optimization and clinical translation.

### 6.5. Neural System Diseases Treatment

The presence of the blood–brain barrier (BBB) restricts the targeted delivery of drugs and cells, resulting in the lack of effective radical treatments for most neurological disorders. In the MC-stem cell system, SCs can differentiate into neurons and glial cells to replace lost neural cells, and secrete neurotrophic factors, anti-inflammatory factors and other cytokines to regulate the pathological microenvironment. In addition, through structural design and functional modification, MCs can provide a 3D microenvironment for SCs, while achieving targeted delivery, long-term survival and precise functional regulation of stem cells, which has become hot in the treatment and repair of neural system diseases including Huntington and stroke.

**Figure 17:**
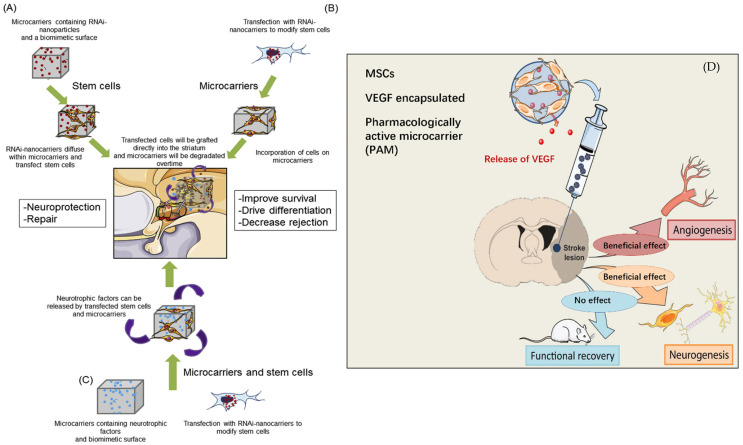
Microcarriers for regulating stem cell fate and treating neural system diseases. (**A**) RNAi-nanocarriers diffuse within microcarriers and transfect stem cells for neuroprotection and neural repair. (**B**) Transfection with RNAi-nanocarriers to modify stem cells and then was incorporated on the microcarriers for neuroprotection and neural repair. (**C**) Microcarriers with biomimetic surface containing neurotrophic factors and stem cells were modified by transfection with RNAi-nanocarriers for neuroprotection and neural repair. Reproduced with permission. [21] 2016, Elsevier (**D**) MSCs attached LM-PAMs microcarriers to treat stroke. Reproduced with permission. [21] 2015, Elsevier.

Huntington’s disease (HD) is an autosomal dominant inherited neurodegenerative disorder. Currently, there is no effective treatment to prevent the occurrence of the disease or stop its progression. At present, research focus is centered on in vivo combined therapeutic strategies, which integrate stem cells, microcarriers, growth factors and epigenetic control of gene expression achieved by different vectors. These strategies aim to improve the engraftment efficiency of stem cells in the brain parenchyma and enhance neuroprotective and neuro-repair capabilities. Figure 17A–C illustrates that microcarriers loaded with RNA interference (RNAi) or neurotrophic factors can spatially regulate stem cell fate, while exerting neuroprotective and neural repair functions [21]. 

Stroke is a major cause of death and disability, with few effective therapeutic interventions to limit brain damage and functional deficits after ischaemic stroke. MSC therapy exerts efficacy through secreting trophic, protective, neurogenic and angiogenic factors, despite the limited survival rate of MSCs. MSCs attached to laminin-pharmacologically active microcarrier (LM-PAMs) was to treat a transient stroke. Results indicated that this microcarrier improved the survival and differentiation of MSCs in Figure 17D. LM-PAMs induced MSCs to express neuronal markers. Conveying MSCs and delivering growth factors via LM-PAMs is an effective strategy for repairing ischaemic stroke-induced brain damage. However, this efficacy is insufficient, at least under the conditions developed in this study [21].

Stem cell-microcarriers have emerged as important agents in the treatment of neurological disorders, with preliminary evidence demonstrated in studies of diseases such as ischemic stroke and Huntington. However, room remains for improving the regulation of stem cell fate and therapeutic efficacy. In addition to optimizing the microcarrier structural design and functional modification to enhance their targeting ability and regulatory precision, efforts should focus on the efficient delivery of microcarrier–stem cell systems across the blood–brain barrier, to provide more effective and safer therapeutic strategies for neurological disorders.

## 7. Conclusions and Outlook

This review provides a systematic elaboration on the regulatory effects of microcarriers on stem cell fate. Based on summarizing advanced fabrication technologies for producing microcarriers with tunable structures and excellent performance, it systematically dissects the core functional mechanisms of the intrinsic static structure parameters (pore size, curvature, surface roughness, microcarrier shape, stiff, hydrophilicity, electrochemical properties and biochemical structure) and dynamic responsive characteristics (pH, ROS levels, thermal stimuli, mechanical signals in the microenvironment) of microcarriers in regulating the directed differentiation, stemness maintenance, and biological functions of stem cells. Finally, we summarize the inherent therapeutic advantages and promising clinical translation potential of functionalized microcarriers in a broad spectrum of biomedical applications, including bone and cartilage defect repair, anti-skin photoaging and psoriasis management, as well as the treatment of ophthalmic, neurodegenerative and autoimmune diseases.

Microcarriers can not only biomimetically reconstruct the in vivo physiological microenvironment of stem cells through precise regulation of the physicochemical properties of materials and functional modification of active extracellular matrix components, thereby achieving high-density expansion of stem cells and stable maintenance of their functional phenotypes, substantially boosting single-batch cell yield, and overcoming the core bottleneck in the scalable manufacturing of clinical-grade stem cell preparations. In addition, as an in vivo delivery and protective carrier for stem cells, microcarriers can effectively resist cell damage induced by in vivo fluid shear stress and host immune clearance after cell transplantation, significantly improve the retention efficiency, survival capacity, and paracrine effects of stem cells at the lesion site, and thus exhibit broad clinical application prospects in the field of stem cell therapy.

However, the industrialization and clinical translation process of microcarrier technology in the stem cell field still faces numerous urgent bottlenecks and limitations to be broken through. To date, GMP-grade compliant biodegradable microcarriers that meet clinical use requirements are limited to only a few categories including gelatin, collagen, poly(lactic-co-glycolic acid) (PLGA), and sodium alginate, leading to an extremely limited range of options. These available materials are difficult to simultaneously satisfy multiple requirements, namely high cell adhesion, controllable degradability, low immunogenicity, inter-batch consistency, and scalable manufacturability. In contrast, novel functionalized microcarriers with excellent performance developed at the laboratory level generally have critical shortcomings such as the lack of toxicological evaluation and long-term biosafety data, as well as immature GMP-grade scalable synthesis processes, which hinder their rapid clinical translation and industrialization. Furthermore, the environmental sensitivity of stem cells brings unmet challenges to shelf-life control and transportation. Specifically, stem cells are intrinsically vulnerable to environmental perturbations: even subtle shifts in temperature, oxygen tension, pH, and osmolarity, paired with mechanical shear forces incurred during transit, can trigger spontaneous differentiation, loss of stemness, diminished viability, and functional heterogeneity—all of which directly erode the therapeutic consistency and efficacy of stem cell products. While conventional DMSO-formulated cryopreservation enables long-term biobanking, it hinges on stringent cold-chain infrastructure and labor-intensive post-thaw recovery protocols, compounding operational complexity and clinical risk. Emerging preservation modalities, including ambient-temperature hydrogel encapsulation and lyophilization, have shown promise for extending product shelf life, yet remain hampered by suboptimal retention of cellular function and a scarcity of large-scale clinical validation. Notably, the absence of standardized transportation quality control frameworks, harmonized stability assessment metrics, and cost-effective end-to-end logistics solutions persists as a core bottleneck impeding the industrial translation of stem cell products.

Looking forward, with the in-depth interdisciplinary integration of biomaterials science, cell engineering, regenerative medicine, and artificial intelligence (AI) technology, microcarriers are expected to mainly develop as follows. First, based on the biomimetic design of the physiological microenvironment of stem cells, we will elucidate the molecular mechanisms underlying material–cell interactions and their combination with other emerging technologies, such as AI, to develop novel functionalized microcarriers integrating intelligent responsive degradation, multi-functional active modification, and high biosafety, thereby achieving precise and controllable regulation of stem cell fate. Second, we will establish an efficient harvesting process for cells adherent to microcarrier surfaces, to achieve complete detachment of stem cells from microcarriers while highly preserving their immunomodulatory properties, stemness, and regenerative potential, and to guarantee the repeatability and production efficiency of the stable expansion process of clinical-grade stem cells. Third, combined with in vivo imaging and lineage-tracing technologies, we will systematically elucidate the in vivo fate regulation rules and therapeutic mechanisms of the microcarrier–stem cell system, screen the microcarrier–stem cell system adapted to specific diseases, and realize the precision and safety of stem cell therapy. Fourth, we will improve the technical standards and regulatory systems for clinical-grade microcarriers and stem cell preparations, and promote the transition of microcarrier-mediated stem cell therapy from individualized clinical exploration to standardized and industrialized clinical application.

## Figures and Tables

**Table 1:** Common commercial microcarriers.

Name	Manufacturer	Material	Surface Coating	Surface Charge
Cytodex-1	GE Healthcare	Dextran/DEAE	None	Positive Charge
Cytopore1,2	GE Healthcare	Cellulose/DEAE	None	Positive Charge
Cytodex-3	GE Healthcare	Dextran/Collagen	None	None
Cultispher-S	Percell-Biolytica	Gelatin	None	None
Hillex II	SoloHill	Polystyrene	None	Positive Charge
FACT III	SoloHill	Polystyrene	None	Positive Charge
PlasticPlus	SoloHill	Polystyrene	None	Positive Charge
Collagen	SoloHill	Polystyrene/Collagen	Collagen	None
Synthemax^®^II	Corning	Polystyrene/Peptide	Peptide	None
DE-52	Whatman	Cellulose/DEAE	None	Positive Charge
SHMC-1225	Zhongke Senhui	Dextran-Glucan	None	—
DASEA Regencarrier^®^	Zhongke Ruiji	Recombinant Human Collagen	None	—
3D RecomTrix^®^	Huakan Biotechnology	Recombinant Collagen	None	—

**Table 2:** Characteristics of various advanced fabrication methods of microcarriers.

Preparation Method	Advantages	Disadvantages
Microfluidics	High controllability of particle size Good monodispersity Precise and controllable structure One-step fabrication of multi-compartment structures	Low production efficiency High fabrication cost Inevitable use of oils, photo initiators, crosslinkers, surfactants, and UV-irradiation
Electrospraying	Precise control of morphology and size Mild fabrication conditions Oil-free and low organic solvent residue	Small-scale controllability Electric field interference in multi-needle arrays Poor processability Easy nozzle clogging
Isothermal spherulitic crystallization	Solvent-free, eco-friendly and biocompatible Independently tunable size and porosity Easy scalable production	Only for crystallizable polymers Process optimization requirements Parameter regulation constraints
Alternating viscous-inertial forces jetting	Ultra-mild, preserves bioink bioactivity Broad biomaterial compatibility Tunable size, excellent cell compatibility	Low fabrication stability Limited driving force Restricted printable ink range
In-air microfluidic techniques	Ultra-high production throughput Oil/emulsifier-free, simplified downstream process Precise size control, narrow distribution	Complex operation, hard scale-up Droplet stability sensitive to environmental parameters Post-processing required
Integrated in situ functionalization fabrication	One-step fabrication and functionalization Uniform distribution High biological activity retention	Complex reaction system design High cost of functional groups Difficulty in large-scale production

## Data Availability

No new data were created or analyzed in this study. Data sharing is not applicable to this article.
